# Kinetic profiling and functional characterization of 8-phenylxanthine derivatives as A_2B_ adenosine receptor antagonists

**DOI:** 10.1016/j.bcp.2022.115027

**Published:** 2022-04-06

**Authors:** Anna Vlachodimou, Henk de Vries, Milena Pasoli, Miranda Goudswaard, Soon-Ai Kim, Yong-Chul Kim, Mirko Scortichini, Melissa Marshall, Joel Linden, Laura H. Heitman, Kenneth A. Jacobson, Adriaan P. IJzerman

**Affiliations:** a Division of Drug Discovery and Safety, Leiden Academic Centre for Drug Research (LACDR), Leiden University, P.O. Box 9502, 2300 RA Leiden, the Netherlands; b Laboratory of Bioorganic Chemistry, National Institute of Diabetes and Digestive and Kidney Diseases, NIH, 9000 Rockville Pike, Bethesda, MD 20892, USA; c Oncode Institute, Leiden, the Netherlands; d Department of Internal Medicine and Molecular Physiology & Biological Physics, University of Virginia Health Science Center, Charlottesville, VA 22908, USA

**Keywords:** A_2B_ adenosine receptor, Antagonists, Binding kinetics, Radioligand binding assay, Label-free functional assay, Covalent binding

## Abstract

A_2B_ adenosine receptor (A_2B_AR) antagonists have therapeutic potential in inflammation-related diseases such as asthma, chronic obstructive pulmonary disease and cancer. However, no drug is currently clinically approved, creating a demand for research on novel antagonists. Over the last decade, the study of target binding kinetics, along with affinity and potency, has been proven valuable in early drug discovery stages, as it is associated with improved *in vivo* drug efficacy and safety. In this study, we report the synthesis and biological evaluation of a series of xanthine derivatives as A_2B_AR antagonists, including an isothiocyanate derivative designed to bind covalently to the receptor. All 28 final compounds were assessed in radioligand binding experiments, to evaluate their affinity and for those qualifying, kinetic binding parameters. Both structure-affinity and structure-kinetic relationships were derived, providing a clear relationship between affinity and dissociation rate constants. Two structurally similar compounds, **17** and **18**, were further evaluated in a label-free assay due to their divergent kinetic profiles. An extended cellular response was associated with long A_2B_AR residence times. This link between a ligand’s A_2B_AR residence time and its functional effect highlights the importance of binding kinetics as a selection parameter in the early stages of drug discovery.

## 1. Introduction

The A_2B_ adenosine receptor (A_2B_AR) belongs to the superfamily of rhodopsin-like G protein-coupled receptors (GPCRs), being a member of the adenosine receptor (AR) family. It has been mapped on chromosome 17p11.2–12, and as all GPCRs the encoded protein consists of a seven transmembrane (7TM) α-helix architecture [1,2]. Adenosine, a ubiquitous purine nucleoside, is the endogenous ligand for all ARs, i.e. A_1_, A_2A_, A_2B_ and A_3_. These AR subtypes are coupled to different effectors and modulate different physiological and pathophysiological conditions. A_2B_AR is the least well characterized of the four AR subtypes, possibly due to its low affinity for adenosine [3]. Under physiological conditions A_2B_AR is considered to remain silent, as the extracellular concentration of adenosine ranges from 20 to 300 nM, much lower than the reported half maximal effective concentration (EC_50_) of adenosine for A_2B_AR (EC_50_ = 24 μM). In contrast, under pathophysiological conditions extracellular concentrations of adenosine could rise up to 30 μM, therefore resulting in A_2B_AR activation and signaling [4,5].

A_2B_ARs are present in numerous tissues and organs, including bowel, bladder, lung, brain, as well as on hematopoietic and mast cells [2,6]. Interestingly, A_2B_AR expression levels are often (up)regulated during disease. The high expression of the receptor in conjunction with the increased extracellular adenosine concentration under pathophysiological conditions render A_2B_AR antagonists interesting pharmacological and therapeutic tools for a broad spectrum of diseases, such as asthma, chronic obstructive pulmonary disease [7,8], colon inflammation [9,10], diabetes [11] and cancer [12]. Adenosine production is upregulated in the tumor microenvironment and acts at both A_2A_AR and A_2B_AR to facilitate tumor progression *in vivo* [13]. In cancer models A_2B_AR antagonists impede adenosine-induced tumor cell proliferation, angiogenesis and metastasis, and remove immune suppression [14].

Over the past years, various xanthine and non-xanthine derivatives have been synthesized and evaluated for their A_2B_AR affinity and selectivity [15–18]. However, no A_2B_AR-selective antagonist has reached the market yet for therapeutic use. Only CVT-6883 has completed Phase I clinical trials with no adverse events reported, while another clinical trial for PBF-1129, as drug candidate for locally advanced or metastatic non-small cell lung carcinoma, is under recruitment [19,20].

The 3D structure of A_2B_AR has not been elucidated yet, hence, the design of new potential drug candidates is mainly based on more classical structure-affinity relationships or on molecular modeling based on homology to the A_2A_AR [21]. Although affinity is a key parameter in pharmacology, it does not necessarily predict *in vivo* efficacy. During the last decade an increasing number of studies suggested that the study of ligand binding kinetics, quantified by association (*k*_*on*_) and dissociation (*k*_*off*_) rate constants, is highly relevant in the early stages of drug discovery, as *in vivo* efficacy is linked to optimized kinetic characteristics in many cases [22]. A typical example is the neurokinin 1 (NK_1_) receptor antagonist aprepitant, an antiemetic. Aprepitant has been found to have higher *in vivo* efficacy than other NK_1_ receptor antagonists with similar thermodynamic affinities, due to its long residence time (RT: 1/*k*_*off*_) at the NK_1_ receptor [23].

Here, we report on the synthesis of a number of xanthine-based A_2B_AR antagonists, on the affinities of these and a number of previously reported xanthines, and on their kinetic target binding parameters obtained in radioligand binding assays. Although not very selective most xanthine derivatives present high affinity for A_2B_AR, while displaying a variety in association and dissociation rate constants. On the basis of these results we also synthesized and pharmacologically profiled compound **29**, a xanthine antagonist that presumably binds covalently to A_2B_AR. Additionally, we developed a label-free impedance-based assay using intact cells expressing A_2B_AR for the further characterization of compounds with diverse kinetic profiles. Compounds **17** and **18** with a long and short RT on the receptor, respectively, were profiled in this assay. Compound **17**, with the longer RT, had a more sustained effect than compound **18**, suggesting this assay has translational relevance.

## 2. Materials and methods

### 2.1. Chemistry

Synthetic reagents and solvents were purchased from Sigma-Aldrich (St. Louis, MO, USA), or prepared as reported. ^1^H NMR spectra were obtained with a Bruker 400 spectrometer using CDCl_3_, DMSO-*d*_6_, acetone-*d*_6_ or CDCl_3_ as a solvent. The chemical shifts are expressed as ppm, and the coupling constants (*J*) are given in Hz. High resolution mass (HRMS) measurements were performed on a proteomics optimized Micromass Q-TOF-2 (Waters, Milford, MA, USA). All chemicals not further specified were from standard commercial sources. Compounds **2**, **8**, **11**–**19**, **26** and **27** were reported in Kim *et al*. [24].

#### 2.1.1. General procedure for the preparation of benzylamide derivatives 3–7, 9, 10, 20–25, and 28

A solution of XCC (**1**, 8-[4-[carboxymethyloxy]phenyl]-1,3-di-(n-propyl)xanthine (1 eq) [25], the desired amine compound (1 eq), EDAC (1-ethyl-3-(2-dimethylaminoethyl)carbodiimide, 2 eq.) and DMAP (4-[N,N-(dimethylamino)]pyridine, 2.2 eq) in 2 mL of anhydrous dimethylformamide was stirred at room temperature for 24 h. The reaction mixture was evaporated to dryness under a stream of nitrogen, and the residue was purified by preparative silica gel thin layer chromatography (chloroform:methanol = 20:1) and crystallization in methanol/ethyl ether to afford the desired compounds.

##### N-cyclopropyl-2-(4-(2,6-dioxo-1,3-dipropyl-2,3,6,7-tetrahydro-1H-purin-8-yl)phenoxy)acetamide (3).

Compound 3 was synthesized following the general procedure using cyclopropilamine and obtaining 15.0 mg of pure compound (68% yield).

^1^H NMR (DMSO, 400 Hz) *δ* 8.17 (d, 1H, *J* = 3.3 Hz), 8.06 (d, 2H, *J* = 8.7 Hz), 7.06 (d, 2H, *J* = 8.7 Hz, 2H), 4.51 (s, 2H), 4.01 (t, 2H, *J* = 7.1 Hz), 3.86 (t, 2H, *J* = 7.3 Hz), 3.87 (t, 2H, *J* = 7.3 Hz), 2.67–2.71 (m, 1H), 1.73 (q, 2H, *J* = 7.4, 14.5 Hz), 1.57 (q, 2H, *J* = 7.4, 14.5 Hz), 0.85–0.92 (m, 6H), 0.61–0.66 (m, 2H), 0.46–0.50 (m, 2H).

HRMS calcd C_22_H_28_N_5_O_4_ (M + H)^+^: 426.2141, found 426.2139.

##### N-cyclobutyl-2-(4-(2,6-dioxo-1,3-dipropyl-2,3,6,7-tetrahydro-1H-purin-8-yl)phenoxy)acetamide (4).

Compound 4 was synthesized following the general procedure using cyclobutylamine and obtaining 19.0 mg of pure compound (95% yield).

^1^H NMR (DMSO + CDCl_3_, 300 Hz) *δ* 8.26 (d, 1H, *J* = 8.1 Hz), 8.60 (d, 2H, *J* = 9.0 Hz), 7.59 (d, 2H, *J* = 9.0 Hz), 4.49 (s, 2H), 4.28 (dd, 1H, *J* = 7.5, 16.2 Hz), 4.20 (t, 2H, *J* = 7.2 Hz), 3.88 (t, 2H, *J* = 7.4 Hz), 2.1–2.2 (m, 2H), 1.94–2.4 (m, 2H), 1.71–1.80 (m, 2H), 1.54–1.71 (m, 4H), 0.92 (t, 3H, *J* = 5.1 Hz), 0.87 (t, 3H, *J* = 5.1 Hz).

HRMS calcd C_24_H_23_N_6_O_4_ (M + H)^+^: 459.1781; found 459.1779.

##### N-cyclopentyl-2-(4-(2,6-dioxo-1,3-dipropyl-2,3,6,7-tetrahydro-1H-purin-8-yl)phenoxy)acetamide (5).

Compound 5 was synthesized following the general procedure using cyclopentylamine and obtaining 20.0 mg of pure compound (85% yield).

^1^H NMR (DMSO, 400 Hz) *δ* 8.06 (d, 2H, *J* = 8.7 Hz), 8.01 (d, 1H, *J* = 5.3 Hz), 7.06 (d, 2H, *J* = 8.8 Hz, 2H), 4.52 (s, 2H), 3.99–4.08 (m, 4H), 3.84 (t, 4H, *J* = 7.4 Hz), 1.40–1.82 (m, 12H), 0.85–0.92 (m, 6H).

HRMS calcd C_24_H_32_N_5_O_4_ (M + H)^+^: 454.2454; found 454.2457.

##### 2-(4-(2,6-dioxo-1,3-dipropyl-2,3,6,7-tetrahydro-1H-purin-8-yl) phenoxy)-N-(1H-pyrazol-3-yl)acetamide (6).

Compound 6 was synthesized following the general procedure using 3-aminopyrazole and obtaining 5.0 mg of pure compound (21% yield).

^1^H NMR (DMSO, 400 Hz) *δ* 12.39 (s, 1H), 10.56 (s, 1H), 8.07 (d, 2H, *J* = 8.7 Hz), 7.62 (s, 1H), 7.09 (d, 2H, *J* = 8.6 Hz, 2H), 6.49 (s, 1H), 4.77 (s, 2H), 4.01 (s, 2H), 3.86 (t, 2H, *J* = 7.2 Hz), 1.72 (q, 2H, *J* = 7.4, 14.5 Hz), 1.57 (q, 2H, *J* = 7.3, 14.4 Hz), 0.85–0.92 (m, 6H).

HRMS calcd C_22_H_26_N_7_O_4_ (M + H)^+^: 452.2046; found 452.2047.

##### N-cyclohexyl-2-(4-(2,6-dioxo-1,3-dipropyl-2,3,6,7-tetrahydro-1H-purin-8-yl)phenoxy)acetamide (7).

Compound 7 was synthesized following the general procedure using cyclohexylamine and obtaining 19.0 mg of pure compound (79% yield).

^1^H NMR (DMSO, 400 Hz) *δ* 8.06 (d, 2H, *J* = 8.7 Hz), 7.92 (d, 1H, *J* = 8.2 Hz), 7.07 (d, 2H, *J* = 8.7 Hz, 2H), 4.52 (s, 2H), 4.01 (t, 2H, *J* = 7.2 Hz), 3.86 (t, 2H, *J* = 7.3 Hz), 3.61 (s, 1H), 1.66–1.76 (m, 5H), 1.55–1.63 (m, 3H), 1.24–1.26 (m, 3H), 1.10–1.12 (m, 1H), 0.85–0.92 (m, 6H).

HRMS calcd C_25_H_34_N_5_O_4_ (M + H)^+^: 468.2611; found 468.2618.

##### 2-(4-(2,6-dioxo-1,3-dipropyl-2,3,6,7-tetrahydro-1H-purin-8-yl) phenoxy)-N-(pyridin-4-ylmethyl)acetamide (9).

Compound 9 was synthesized following the general procedure using 4-(methylamino) pyridine and obtaining 11.0 mg of pure compound (45% yield).

^1^H NMR (DMSO, 400 Hz) *δ* 8.79 (t, 1H, *J* = 5.7 Hz), 8.47 (d, 2H, *J* = 5.6 Hz), 8.08 (d, 2H, *J* = 8.8 Hz), 7.23 (d, 1H, *J* = 5.5 Hz), 7.12 (d, 2H, *J* = 8.7 Hz, 2H), 4.68 (s, 2H), 4.37 (d, 2H, *J* = 6.1 Hz), 4.02 (t, 2H, *J* = 7.2 Hz), 3.87 (t, 2H, *J* = 7.0 Hz), 1.74 (q, 2H, *J* = 7.4, 14.5 Hz), 1.58 (q, 2H, *J* = 7.3, 14.4 Hz), 0.85–0.92 (m, 6H).

HRMS calcd C_25_H_29_N_6_O_4_ (M + H)^+^: 477.2250; found 477.2243.

##### 2-(4-(2,6-dioxo-1,3-dipropyl-2,3,6,7-tetrahydro-1H-purin-8-yl) phenoxy)-N-(pyrazin-2-yl)acetamide (10).

Compound 10 was synthesized following the general procedure using aminopyrazine and obtaining 6.5 mg of pure compound (27% yield).

^1^H NMR (DMSO, 400 Hz) *δ* 10.97 (s, 1H), 9.30 (s, 1H), 8.44 (s, 1H), 8.40 (s, 1H), 8.07 (d, 2H, *J* = 8.7 Hz), 7.11 (d, 2H, *J* = 8.7 Hz, 2H), 4.94 (s, 2H), 2.38 (t, 2H, *J* = 7.1 Hz), 3.86 (t, 2H, *J* = 7.2 Hz), 1.73 (q, 2H, *J* = 7.4, 14.5 Hz), 1.58 (q, 2H, *J* = 7.3, 14.4 Hz), 0.85–0.92 (m, 6H).

HRMS calcd C_23_H_26_N_7_O_4_ (M + H)^+^: 464.2046; found 464.2047.

##### 2-(4-(2,6-dioxo-1,3-dipropyl-2,3,6,7-tetrahydro-1H-purin-8-yl) phenoxy)-N-(4-methylbenzyl)acetamide (20).

Compound 20 was synthesized following the general procedure using 4-methylbenzylamine and obtaining 44.5 mg of pure compound (91% yield).

^1^H NMR (DMSO, 300 Hz) *δ* 8.64 (t, 1H, *J* = 5.9 Hz), 8.07 (d, 2H, *J* = 8.8 Hz), 7.16 − 7.08 (m, 6H), 4.63 (s, 2H), 4.30 (d, 2H, *J* = 5.9 Hz), 4.02 (t, 2H, *J* = 7.0 Hz), 3.87 (t, 2H, *J* = 7.3 Hz), 2.26 (s, 3H), 1.74 (m, 2H), 1.58 (m, 2H), 0.91 (t, 3H, *J* = 7.6 Hz), 0.88 (t, 3H, *J* = 7.7 Hz).

HRMS calcd C_27_H_32_N_5_O_4_ (M + H)^+^: 490.2454; found 490.2462.

##### 2-(4-(2,6-dioxo-1,3-dipropyl-2,3,6,7-tetrahydro-1H-purin-8-yl) phenoxy)-N-(4-fluorobenzyl)acetamide (21).

Compound 21 was synthesized following the general procedure using 4-fluorobenzylamine and obtaining 32.3 mg of pure compound (65% yield).

^1^H NMR (DMSO, 300 Hz) *δ* 8.71 (t, 1H, *J* = 5.9 Hz), 8.07 (d, 2H, *J* = 8.8 Hz), 7.30 (dd, 2H, *J* = 5.9, 8.5 Hz), 7.16−7.08 (m, 4H), 4.64 (s, 2H), 4.33 (d, 2H, *J* = 6.0 Hz), 4.02 (t, 2H, *J* = 7.1 Hz), 3.87 (t, 2H, *J* = 7.3 Hz), 1.74 (m, 2H), 1.58 (m, 2H), 0.91 (t, 3H, *J* = 7.6 Hz), 0.88 (t, 3H, *J* = 7.6 Hz).

HRMS calcd C_26_H_29_FN_5_O_4_ (M + H)^+^: 494.2204; found 494.2199.

##### N-(4-bromobenzyl)-2-(4-(2,6-dioxo-1,3-dipropyl-2,3,6,7-tetrahydro-1H-purin-8-yl)phenoxy)acetamide (22).

Compound 22 was synthesized following the general procedure using 4-bromobenzylamine and obtaining 30.0 mg of pure compound (54% yield).

^1^H NMR (DMSO, 300 Hz) *δ* 8.75 (t, 1H, *J* = 6.1 Hz), 8.09 (d, 2H, *J* = 8.7 Hz), 7.47−7.40 (m, 2H), 7.27 (d, 2H, *J* = 4.9 Hz), 7.10 (d, 2H, *J* = 8.9 Hz), 4.66 (s, 2H), 4.35 (d, 2H, *J* = 6.1 Hz), 4.02 (t, 2H, *J* = 7.1 Hz), 3.87 (t, 2H, *J* = 7.3 Hz), 1.74 (m, 2H), 1.58 (m, 2H), 0.91 (t, 3H, *J* = 7.5 Hz), 0.88 (t, 3H, *J* = 7.6 Hz).

HRMS calcd C_26_H_29_BrN_5_O_4_ (M + H)^+^: 554.1403; found 554.1397.

##### N-(3,4-dihydroxybenzyl)-2-(4-(2,6-dioxo-1,3-dipropyl-2,3,6,7-tetrahydro-1H-purin-8-yl)phenoxy)acetamide (23).

Compound 23 was synthesized following the general procedure using 3,4-dihydroxybenzylamine hydrobromide and obtaining 18.7 mg of pure compound (37% yield).

^1^H NMR (DMSO, 300 Hz) *δ* 8.53 (t, 1H, *J* = 6.0 Hz), 8.06 (d, 2H, *J* = 8.9 Hz), 7.08 (d, 2H, *J* = 8.8 Hz), 6.69 (d, 1H, *J* = 1.8 Hz), 6.65 (d, 1H, *J* = 8.0 Hz), 6.51 (dd, 1H, *J* = 8.1, 1.9 Hz), 4.59 (s, 2H), 4.17 (d, 2H, *J* = 6.0 Hz), 4.01 (t, 2H, *J* = 7.2 Hz), 3.86 (t, 2H, *J* = 7.3 Hz), 1.74 (m, 2H), 1.58 (m, 2H), 0.90 (t, 3H, *J* = 7.4 Hz), 0.87 (t, 3H, *J* = 7.6 Hz).

HRMS calcd C_26_H_30_N_5_O_6_ (M + H)^+^: 508.2196; found 508.2184.

##### (R)-2-(4-(2,6-dioxo-1,3-dipropyl-2,3,6,7-tetrahydro-1H-purin-8-yl)phenoxy)-N-(1-phenylethyl)acetamide (24).

Compound 24 was synthesized following the general procedure using R-(+)-α-methylbenzylamine and obtaining 28.6 mg of pure compound (58% yield).

^1^H NMR (DMSO, 300 Hz) *δ* 8.57 (d, 1H, *J* = 8.4 Hz), 8.06 (d, 2H, *J* = 8.7 Hz), 7.33−7.20 (m, 5H), 7.08 (d, 2H, *J* = 8.7 Hz), 5.01 (m, 1H), 4.61 (s, 2H), 4.02 (t, 2H, *J* = 7.1 Hz), 3.87 (t, 2H, *J* = 7.3 Hz), 1.74 (m, 2H), 1.58 (m, 2H), 1,41 (d, 3H, *J* = 6.9 Hz), 0.90 (t, 3H, *J* = 7.6 Hz), 0.88 (t, 3H, *J* = 7.8 Hz).

HRMS calcd C_27_H_32_N_5_O_4_ (M + H)^+^: 490.2454; found 490.2433.

##### (S)-2-(4-(2,6-dioxo-1,3-dipropyl-2,3,6,7-tetrahydro-1H-purin-8-yl)phenoxy)-N-(1-phenylethyl)acetamide (25).

Compound 25 was synthesized following the general procedure using S-(-)-α-methylbenzylamine and obtaining 22.0 mg of pure compound (45% yield).

^1^H NMR (DMSO, 300 Hz) *δ* 8.57 (d, 1H, *J* = 8.1 Hz), 8.06 (d, 2H, *J* = 8.7 Hz), 7.33−7.20 (m, 5H), 7.08 (d, 2H, *J* = 8.7 Hz), 5.01 (m, 1H), 4.61 (s, 2H), 4.02 (t, 2H, *J* = 7.1 Hz), 3.87 (t, 2H, *J* = 7.2 Hz), 1.74 (m, 2H), 1.58 (m, 2H), 1,41 (d, 3H, *J* = 7.0 Hz), 0.90 (t, 3H, *J* = 7.6 Hz), 0.88 (t, 3H, *J* = 7.7 Hz).

HRMS calcd C_27_H_32_N_5_O_4_ (M + H)^+^: 490.2454; found 490.2429.

##### General procedure for the preparation of compound 29.

N-(4-Aminophenyl)-2-(4-(2,6-dioxo-1,3-dipropyl-2,3,6,7-tetrahydro-1H-purin-8-yl)phenoxy)acetamide (**28**) was prepared reacting XCC with p-phenylenediamine in the presence of EDAC and DMAP as carboxyl group activating agents. Due to the chemical sensitivity of this particular p-amino-anilide bond, after work-up of the reaction mixture, compound **28** was directly used in the successive step without further purification. Reaction of the latter with thiophosgene furnished compound **29** (2-(4-(2,6-dioxo-1,3-dipropyl-2,3,6,7-tetrahydro-1H-purin-8-yl)phenoxy)-N-(4-isothiocyanatophenyl)acetamide).

##### N-(4-Aminophenyl)-2-(4-(2,6-dioxo-1,3-dipropyl-2,3,6,7-tetrahydro-1H-purin-8-yl)phenoxy)acetamide (28).

A solution of XCC (0.06 mmol, **1**), p-phenylenediamine (0.06 mmol), EDAC (0.130 mmol), and DMAP (0.130 mmol) in 1 mL of anhydrous DMF was stirred at room temperature for 18 h. The following day, a sample of the solution was withdrawn for MS-LC analysis showing complete conversion to compound **28**. Water (2 mL) was added to the solution with formation of a white solid that was filtered and washed three times with ether (26 mg, 90 %).

HRMS calcd C_25_H_29_N_6_O_4_ (M + H)_+_: 477.2316; found 477.2311.

##### 2-(4-(2,6-dioxo-1,3-dipropyl-2,3,6,7-tetrahydro-1H-purin-8-yl) phenoxy)-N-(4-isothiocyanatophenyl)acetamide (29).

To a suspension of **28** (0.05 mmol) in 8 mL of CHCl_3_ and NaHCO_3_ saturated water solution (3:1) was added thiophosgene (0.33 mmol) all at once under vigorous stirring, and the mixture stirred at room temperature for 5 min. Then, additional CHCl_3_ (20 mL) and water (5 mL) were added to break the emulsion. The phases were separated, and the aqueous phase was washed with CHCl_3_ (3 × 25 mL). The organic phases were combined, dried over Na_2_SO_4_ and the solvent removed under vacuum to obtain 6.6 mg (25% yield) of the pure compound **29**.

^1^H NMR (CDCl_3_, 400 Hz) *δ* 10.5. (s, 1H), 8.20 (d, *J* = 0.02 Hz, 2H), 7.82 (d, *J* = 0.02 Hz, 2H), 7.51 (d, *J* = 0.02 Hz, 2H), 7.21 (d, *J* = 0.02 Hz, 2H), 4.90 (s, 2H), 4.13 (t, *J* = 0.02 Hz, 2H), 3.95 (t, *J* = 0.02 Hz, 2H), 1.85 (q, *J* = 0.03 Hz, 2H), 1.48 (q, *J* = 0.03 Hz, 2H), 0.90–1.09 (m, 4H).

### 2.2. Biology

#### 2.2.1. Chemicals and reagents

Bovine serum albumin (BSA) and the bicinchoninic acid (BCA) protein assay kit were purchased from Fisher Scientific (Hampton, New Hampshire, United States). [^3^H]PSB-603 (specific activity 79 Ci mmol^−1^) was custom-labeled and purchased from Quotient Bioresearch (Waltham, MA) and ZM241385 was a kind gift by Zeneca Pharmaceuticals (Macclesfield, United Kingdom). Adenosine deaminase (ADA) was purchased from Sigma Aldrich (Zwijndrecht, the Netherlands). All other chemicals used in the biological experiments were purchased from standard commercial sources.

CHO-spap cells either “empty” or stably expressing the wildtype human A_2B_AR (CHO-spap-hA_2B_AR) were kindly provided by S.J. Dowell, Glaxo Smith Kline.

#### 2.2.2. Cell culture

CHO-spap cells were grown in Dulbecco’s modified Eagle’s medium: Nutrient Mixture F-12 (DMEM/F12) supplemented with 10% (v/v) newborn calf serum, 100 IU/mL penicillin and 100 μg/mL streptomycin at 37 °C and 5% CO_2_. Cells were subcultured at a ratio of 1:20 twice weekly. CHO-spap-hA_2B_AR cells were grown in the same medium supplemented with 1 mg/mL G418 and 0.4 mg/mL hygromycin. Cells were subcultured at a ratio of 1:20 twice weekly.

#### 2.2.3. Membrane preparation

CHO-spap-hA_2B_AR cells were cultured as a monolayer in 15 cm ø plates to about 90% confluency. Cells were removed from the plates by scraping into 5 mL of phosphate-buffered saline (PBS) and centrifuged for 5 min at 1500 rpm. The resulting pellets were resuspended in ice-cold Tris-HCl buffer (50 mM Tris-HCl, pH 7.4) and homogenized using an Ultra Turrax homogenizer (IKA Werke GmbH & Co.KG, Staufen, Germany). Centrifugation at 31,000 rpm in an Optima LE-80 K ultracentrifuge with Ti-70 rotor (Beckman Coulter, Fullerton, CA) at 4 °C for 20 min, resulted in separation of membranes and cytosolic fraction. Subsequently, pellet was resuspended in 10 mL Tris-HCl buffer, homogenized and centrifuged once again. The final pellet was suspended in assay buffer (50 mM Tris-HCl buffer, 0.1% (w/v) CHAPS, pH 7.4), ADA was added to break down endogenous adenosine, and the homogenization step was repeated. Aliquots were stored at −80°C and the membrane protein concentration was determined by a BCA protein determination assay [26]. The BCA results were measured in a Wallac EnVision 2104 Multilabel Reader (Perkin Elmer, Groningen, The Netherlands).

#### 2.2.4. Radioligand binding assay

In all radioligand binding experiments, CHO-spap-hA_2B_AR membranes were thawed and homogenized using an Ultra Turrax homogenizer at 24,000 rpm (IKA-Werke GmbH & Co.KG, Staufen, Germany), diluted in assay buffer to the desired concentration (10–30 μg per well or Eppendorf tube). All materials were brought to 25 °C, 30 min prior to the experiment. ZM241385 (10 μM) was used to determine nonspecific binding (NSB). DMSO concentrations were 2% for all compounds except for **8**, **12**–**18** and **20**–**22**, where the concentration was 0.25%. The two different DMSO concentrations had negligible effects on the radioligand binding results. Finally, total radioligand binding (TB) did not exceed 10% of the [^3^H]PSB-603 present in the assay in order to prevent ligand depletion.

##### 2.2.4.1. Displacement experiments.

Were performed using 1.5 nM [^3^H] PSB-603 and a competing unlabeled ligand at multiple concentrations diluted in assay buffer (50 mM Tris-HCl, 0.1% (w/v) CHAPS, pH 7.4). Binding was initiated by addition of CHO-spap-hA_2B_AR membrane aliquots to reach a total volume of 100 μL. Samples were incubated at 25 °C for 2 h to reach equilibrium. The incubation was terminated by rapid vacuum filtration over 96-well Whatman GF/C filter plates using a PerkinElmer Filtermate harvester (PerkinElmer, Groningen, Netherlands). Filters were subsequently washed ten times using ice-cold wash buffer (50 mM Tris-HCl, 0.1% (w/v) BSA, pH 7.4). Filter plates were dried at 55 °C for about 45 min and afterwards 25 μL Microscint (PerkinElmer) was added per well. Filter-bound radioactivity was determined by liquid scintillation spectrometry using a 2450 Microbeta^2^ scintillation counter (PerkinElmer).

##### 2.2.4.2. Saturation binding experiments.

Were carried out by incubating increasing concentrations of [^3^H]PSB-603 (from 0.05 to 5 nM) with membrane aliquots for 2 hr at 25 °C. Non-specific binding was assessed by three concentrations of the radioligand (0.05 nM, 1 nM and 5 nM) and analyzed by linear regression. Incubation was terminated by filtration through GF/C filters using a Brandel-harvester (Brandel Harvester 24w, Gaithersburg, MD, USA). Filters were washed three times using ice-cold wash buffer and collected in tubes. 3.5 mL Emulsifier-Safe scintillation fluid (Perkin Elmer, Groningen, the Netherlands) was added and the filter-bound radioactivity was determined in a Tri-Carb 2900TR liquid scintillation analyzer (PerkinElmer).

##### 2.2.4.3. Association experiments.

Were performed by incubation of [^3^H] PSB-603 (1.5 nM) with membrane aliquots at 25 °C. The amount of receptor-bound radioligand was determined after filtration at different time intervals for a total incubation time of 45 min and samples were obtained as described under “Displacement experiments”.

##### 2.2.4.4. Dissociation experiments.

Were carried out after a 45 min pre-incubation of 1.5 nM [^3^H]PSB-603 and membrane aliquots. Subsequently, dissociation of the radioligand at different time points up to 150 min was initiated by addition of 5 μL ZM241385 (assay concentration 10 μM). Dissociation experiments were performed at 25 °C. The amount of receptor-bound radioligand was determined after filtration and samples were obtained as described under “Displacement experiments”.

##### 2.2.4.5. Competition association experiments.

Were performed at 25 °C and in a total volume of 100 μL, by incubation of 1.5 nM [^3^H]PSB-603 and a competing ligand diluted in assay buffer to reach IC_50_ concentration. Addition of CHO-spap-hA_2B_AR membrane aliquots initiated the association. The amount of receptor-bound radioligand was determined at different time points up to 3 hr. After 3 hr the incubation was terminated and samples were obtained as described under “Displacement experiments”.

##### 2.2.4.6. Washout experiments.

Were performed at 25 °C and in a total volume of 400 μL. CHO-spap-hA_2B_AR membranes were incubated for 2 hr with the unlabeled compounds (at a final concentration of 10 × IC_50_) while shaking at 1,000 RPM in an Eppendorf thermomixer comfort (Eppendorf AG, Hamburg, Germany). Subsequently, the samples were centrifuged at 13200 rpm (16 100g) at 4 °C for 5 min and the supernatant containing unbound ligand was removed. Pellets were resuspended in 1 mL of assay buffer, and samples were incubated for 10 min at 25 °C in the thermomixer. After four centrifugation and washing cycles in total, supernatant was discarded and the membranes were resuspended in a total volume of 400 μL containing 1.5 nM [^3^H]PSB-603. After 2 hr at 25 °C incubations were terminated by rapid filtration through GF/C filters using a Brandel harvester and the samples were obtained as described under “Saturation binding experiments”.

#### 2.2.5. Label-free whole-cell assay

##### 2.2.5.1. Detection principle.

Label-free assays were performed using the xCELLigence real-time cell analyser (RTCA) system [27,28], as described previously [29]. In short, cells attached to the gold-coated electrodes embedded on the bottom of E-plates are generating electrical impedance which is monitored by the RTCA system. Variations in cell number, adhesion, viability and morphology result in impedance changes (Z) which are constantly recorded at 10 kHz. Z is displayed in the unitless parameter called Cell Index (CI) [28,30], which is defined as (Z_i_-Z_0_) Ω /15 Ω. Z_i_ is the impedance at a given time point and Z_0_ represents the baseline impedance in the absence of cells, which is measured prior to the start of the experiment.

##### 2.2.5.2. Determination of pEC_50_ value of NECA for hA_2B_AR.

CHO-spap and CHO-spap-hA_2B_AR cells were harvested and centrifuged at 200x *g* (1500 rpm) for 5 min. Z_0_ was measured in the presence of 45 μL culture media in 96 well PET E-plates (Bioké, Leiden, The Netherlands). 60,000 cells were seeded in a volume of 50 μL per well. After maintaining at room temperature for about 30 min, the E-plate was placed into the recording station housed in a 37 °C and 5% CO_2_ incubator. Impedance was measured every 15 min. After about 19 hr 30 min of recording, cells were treated with NECA (10^−11^ till 10^−6^ M) or vehicle control (0.25% DMSO) in 5 μL. CI was recorded for 90 min (recording schedule: 15 s intervals for 25 min, followed by 1 min intervals for 20 min and 5 min intervals for 45 min).

##### 2.2.5.3. Validation that NECA signaling is hA_2B_AR-mediated.

Z_0_ was measured in the presence of 45 μL culture media in 96 well PET E-plates (Bioké, Leiden, The Netherlands). 60,000 CHO-spap or CHO-spaphA_2B_AR cells were seeded in a volume of 50 μL per well. The E-plate was left for about 30 min at room, and afterwards was placed into the recording station housed in a 37 °C and 5% CO_2_ incubator. Impedance measurements were recorded every 15 min. After about 19 hr 30 min of recording, cells were pre-treated with saturating concentrations of an AR antagonist, i.e. A_1_: DPCPX (45 nM); A_2A_: SCH442416 (4.8 nM); A_2B_: PSB603 (55 nM); A3: PSB11 (230 nM) or vehicle control (vehicle 1; 0.25% DMSO) in 5 μL. CI was recorded for 4 hr (recording schedule: 15 s intervals for 10 min, followed by 1 min intervals for 50 min and 15 min interval for 180 min). Subsequently, cells were treated with NECA (EC_80_) or vehicle control (vehicle 2; 0.25% DMSO) in 5 μL and CI was recorded for 90 min (recording schedule: 15 s intervals for 25 min, followed by 1 min intervals for 15 min and 5 min intervals for 50 min).

##### 2.2.5.4. Determination of pIC_50_ values of hA_2B_AR antagonists.

Z_0_ was measured in the presence of 40 μL culture media in 96 well PET E-plates (Bioké, Leiden, The Netherlands). CHO-spap-hA_2B_AR cells were seeded in a density of 60,000 cells per well (50 μL). After staying 30 min without agitation at room temperature, the E-plate was placed into the recording station housed in a 37 °C and 5% CO_2_ incubator. Impedance was measured every 15 min. After about 19 hr 30 min of recording, cells were pre-treated with A_2B_AR antagonists (10^−9^ till 10^−5^ M) or vehicle control (vehicle 1; 0.25% DMSO) in 5 μL. CI was recorded for 4 hr (recording schedule: 15 s intervals for 10 min, followed by 1 min intervals for 50 min and 15 min interval for 180 min). Subsequently, cells were treated with NECA (EC_80_) or vehicle control (vehicle 2; 0.25% DMSO) in 5 μL and CI was recorded for 90 min (recording schedule: 15 s intervals for 25 min, followed by 1 min intervals for 15 min and 5 min intervals for 50 min).

##### 2.2.5.5. Washout assay.

The assay followed the same initial steps as described in “Determination of pIC_50_ values for hA_2B_AR antagonists”. After about 19 hr 30 min of recording, cells were pre-treated with A_2B_AR antagonists (30 × IC_50_; based on the pIC_50_ value determined with “*Determination of pIC*_*50*_ *values for hA*_*2B*_*AR antagonists”*) or vehicle control (vehicle 1; 0.25% DMSO) in 5 μL. CI was recorded for 4 hr (recording schedule: 15 s intervals for 10 min, followed by 1 min intervals for 50 min and 15 min intervals for 180 min). Subsequently, wells were washed by aspiration of the medium, followed by the addition of 95 μL fresh serum free medium. For the unwashed cells, the medium was not removed but was pipetted up and down to simulate any mechanical cell stress. The E-plate was placed on the recording station and CI was recorded for 30 min (recording schedule: 15 s intervals for 25 min, followed by 1 min intervals for 5 min). Finally, cells were treated with NECA (EC_80_) or vehicle control (vehicle 2; 0.25% DMSO) in 5 μL and CI was recorded for 90 min (recording schedule: 15 s intervals for 25 min, followed by 1 min intervals for 15 min and 5 min intervals for 50 min).

### 2.3. Data analysis

#### 2.3.1. Radioligand binding assays

Data analyses were performed using GraphPad Prism 8.0 software (GraphPad Software Inc., San Diego, CA, USA). For saturation assays, K_D_ and B_max_ values were determined by non-linear regression curve fitting using the one site: “total and non-specific binding” equation. For displacement assays, pIC_50_ values were obtained by non-linear regression curve fitting to a sigmoidal concentration–response curve using the “log(inhibitor) vs. response” GraphPad analysis equation. pK_i_ values were converted from pIC_50_ values and the saturation K_D_ using the Cheng–Prusoff equation [31]:

$${\text{K}}_{\text{i}}={\text{ IC}}_{50}/\left(1+\left[\text{radioligand}\right]/{\text{K}}_{\text{D}}\right)$$

The *k*_*off*_ value was obtained using a one-phase exponential decay analysis of data resulting from a radioligand dissociation assay. The value of *k*_*on*_ was determined using the equation:

$$k_{on}=(k_{obs}–k_{off})/\left[\text{radioligand}\right]$$

in which *k*_*obs*_ was determined using a one phase association analysis of data from a radioligand association assay. The association and dissociation rate constants were used to calculate the kinetic *K*_*D*_ value using: *K*_*D*_ = *k*_*off*_ / *k*_*on*_.

Association and dissociation rate constants for unlabelled A_2B_AR inhibitors were determined by nonlinear regression analysis of competition association data as described by Motulsky and Mahan [32]. The data were fitted into the GraphPad “kinetics of competitive binding” analysis, where *k*_*1*_ and *k*_*2*_ are the *k*_*on*_ (M^−1^ min^−1^) and *k*_off_ (min^−1^) of [^3^H]PSB-603 obtained from radioligand association and dissociation assays, respectively, L is the radioligand concentration (nM), I is the concentration of unlabeled competitor (nM), X is the time (min) and Y is the specific binding of the radioligand (DPM). Fixing these parameters resulted in the calculation of the following parameters: *k*_3_, which is the *k*_on_ value (M^−1^min^−1^) of the unlabeled ligand; *k*_4_, which is the *k*_off_ value (min^−1^) of the unlabeled ligand and B_max_, that equals the total binding (DPM). All competition association data were globally fitted. The residence time (RT) was calculated using RT = 1 / *k*_*off*_ [33].

All values are shown as mean ± S.E.M. of at least three independent experiments performed in duplicate. Statistical analysis was performed if indicated, using a one-way ANOVA with Dunnett’s post-test (**** *P <*0.0001; *** *P <* 0.001; ** *P <* 0.01; * *P <* 0.05) or an unpaired Student’s *t* test (^####^ *P <* 0.0001; ^###^ *P <* 0.001; ^##^ *P <* 0.01; ^#^ *P <* 0.05). Observed differences were considered statistically significant if P-values were below 0.05.

#### 2.3.2. Label-free whole-cell assay

RTCA software 2.0 (ACEA Biosciences, Inc.) was used to record all experimental data. Data were analyzed using GraphPad Prism 8.0. Compound responses, baseline-corrected to vehicle control, were normalized at the time of ligand addition to obtain Normalized Cell Index (NCI) values to correct for compound-independent responses. The time of normalization was either at approximately 19 hr 30 min, at 23 hr 30 min or at 24 hr after cell seeding for analysis of NECA response depending on the type of assay (number of steps), i.e. pre-treatment with antagonists, washing and NECA treatment, respectively.

The absolute values of Area Under the Curve (AUC) up to 90 min after NECA addition were exported from the RTCA software to Graphpad Prism 8.0 for further analysis yielding concentration–response curves. The pEC_50_ value of NECA (Table 5) was obtained using non-linear regression curve fitting of AUC data into “log(agonist) vs. response (three parameters)” analysis. pIC_50_ values of hA_2B_AR antagonists (Table 5) were obtained using non-linear regression curve fitting of AUC data into “log(inhibitor) vs. response (three parameters)” analysis. Data shown are the mean ± S.E.M of at least three individual experiments performed in duplicate.

## 3. Results

### 3.1. Chemistry

**Scheme 1.**
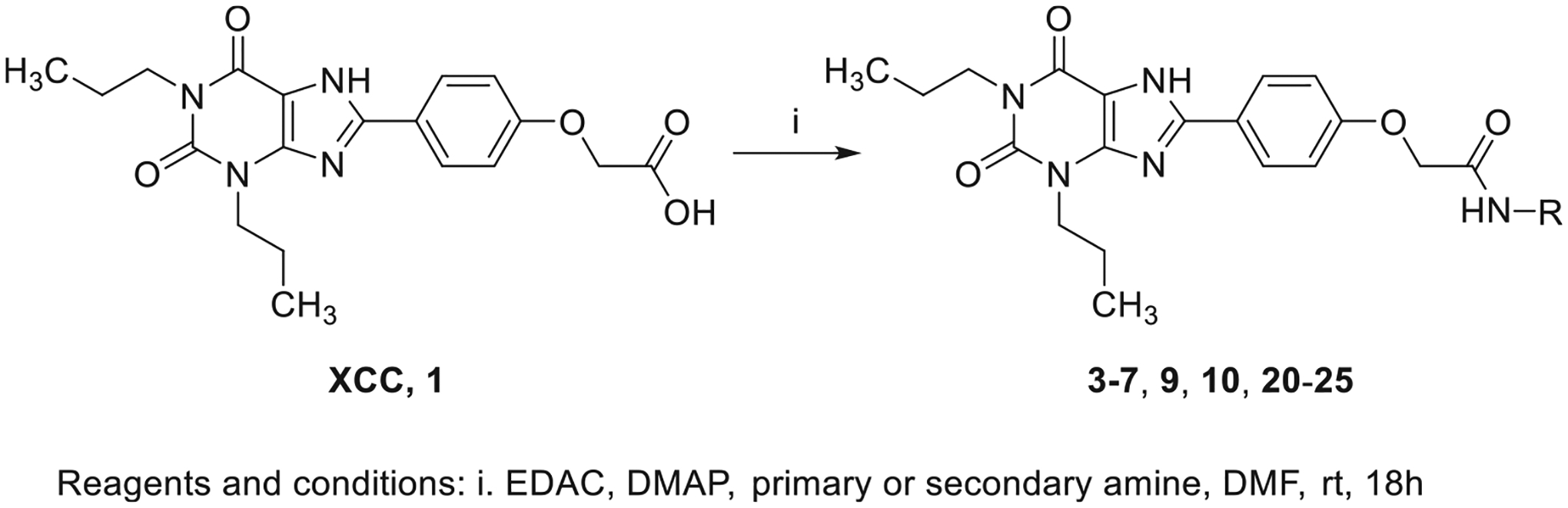
Synthesis of XCC amides.

**Scheme 2.**
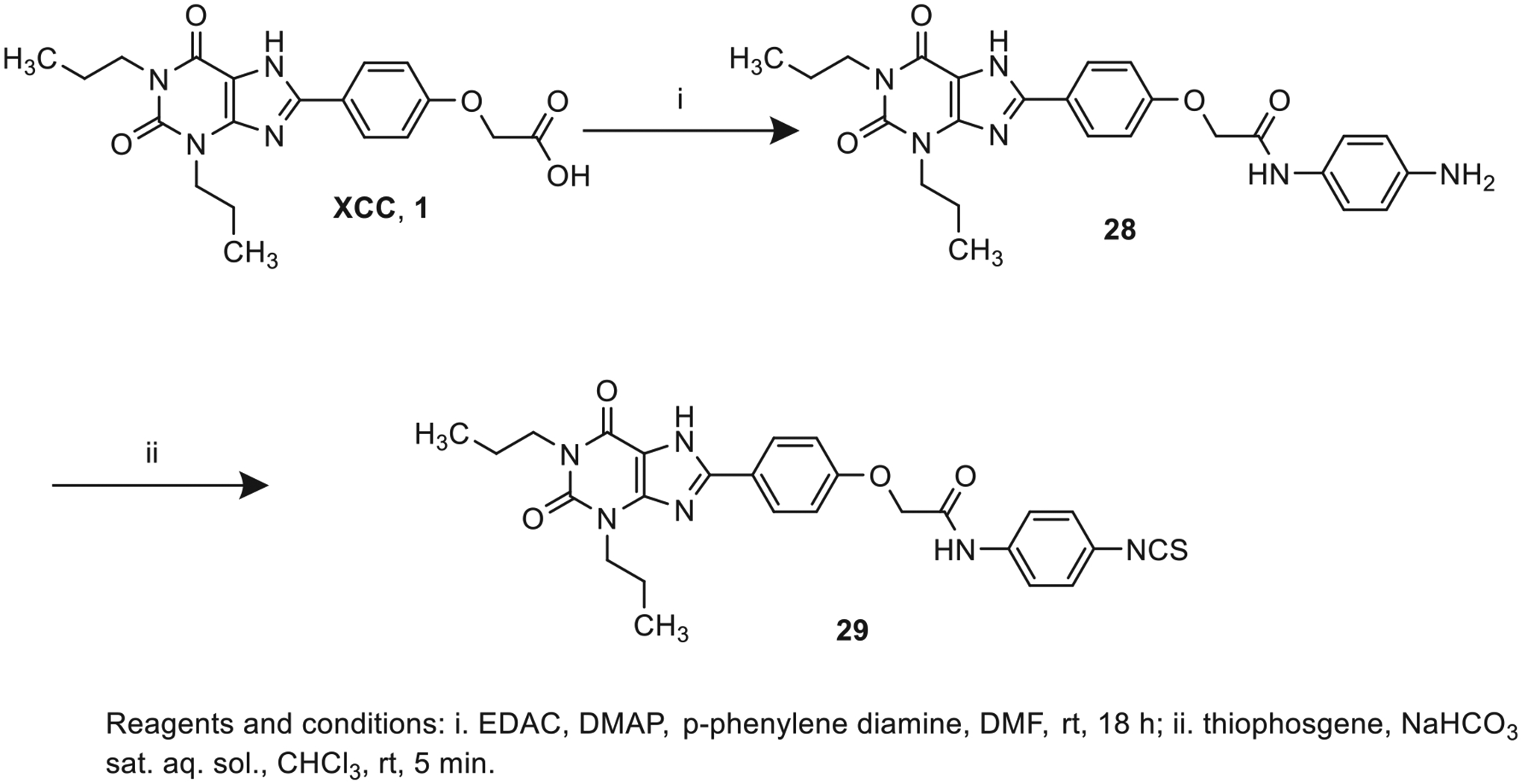
Synthesis of compound **29**, designed as covalently binding antagonist.

2-(4-(2,6-Dioxo-1,3-dipropyl-2,3,6,7-tetrahydro-1H-purin-8-yl)phenoxy)acetic acid (xanthine carboxylic congener, **XCC**, **1**) was synthesized as reported [24]. Its amide derivatives **3**–**7**, **9**, **10**, **20**–**25** were prepared by reaction with the desired amine in the presence of EDAC and DMAP as carboxyl group activating agents (Scheme 1). XCC was also used as the starting reagent for the synthesis of isothiocyanate-containing **29**, aimed to bind to the A_2B_AR covalently (Scheme 2).

### 3.2. Biological Evaluation

#### 3.2.1. Validation of [^3^H]PSB-603 equilibrium and kinetic radioligand binding assays

**Fig. 1.**
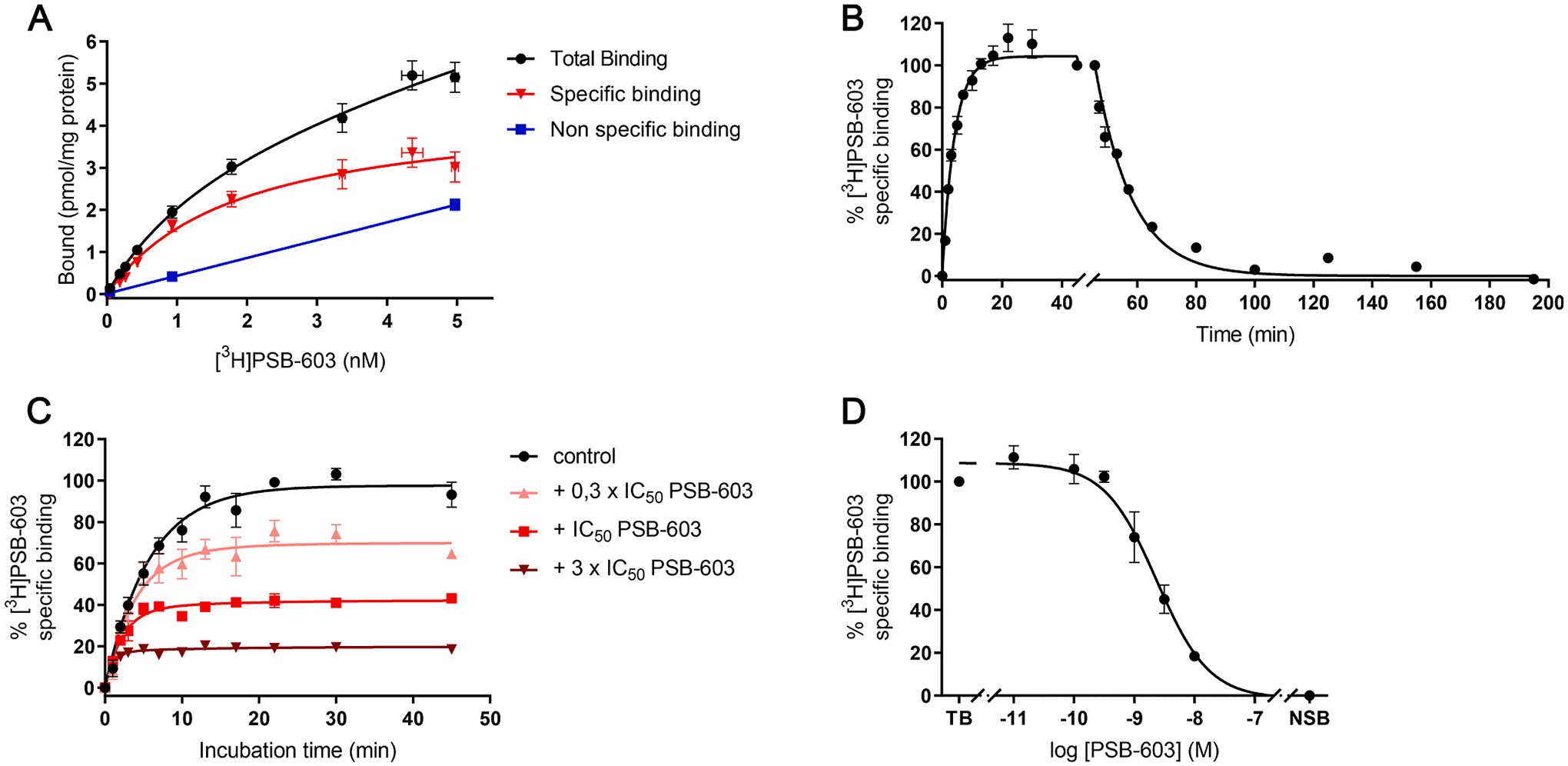
Characterization of [^3^H]PSB-603 binding to hA_2B_AR expressed on CHO-spap-hA_2B_AR membranes at 25 °C. (A) Binding of [^3^H]PSB-603 in an equilibrium saturation assay. (B) Association and dissociation kinetics of 1.5 nM [^3^H]PSB-603 to and from hA_2B_AR (100% equals to approx. 1700 dpm). (C) Competition association assay of [^3^H]PSB-603 in the absence or presence of 0.3x, 1x, and 3x IC_50_ of unlabeled PSB-603 (100% equals to approx. 1700 dpm). (D) Homologous displacement of [^3^H]PSB-603 from hA_2B_AR (100% equals to approx. 1900 dpm). Data are shown as mean ± S.E.M. from at least three independent experiments performed in duplicate.

Firstly, the binding profile of tritium-labeled A_2B_AR antagonist 8-[4-[4-(4-chlorophenzyl)piperazide-1-sulfonyl) phenyl]]-1-propylxanthine ([^3^H]PSB-603) was characterized on CHO-spap-hA2B membranes. In a saturation binding assay receptor binding was saturable and quantified by a K_D_ value of 1.71 nM and a B_max_ value of 4.30 pmol/mg (Fig. 1A, Table 1). When evaluated in a homologous displacement assay, unlabeled PSB-603 showed similar affinity, yielding a pK_i_ value of 8.90 (Fig. 1D, Table 1).

Subsequently, [^3^H]PSB-603 was evaluated in kinetic binding assays in order to determine its kinetic binding parameters *k*_*on*_ and *k*_*off*_ (Fig. 1B, Table 1). [^3^H]PSB-603 associated rapidly to the hA_2B_AR and equilibrium binding was reached within 20 min, while complete dissociation was reached within 55 min, resulting in a *k*_*off*_ value of 0.075 min^−1^ The association and dissociation experiments resulted in the calculation of *k*_*on*_ and RT values of 0.096 nM^−1^ min^−1^ and 13 min, respectively. Based on the kinetic data a dissociation constant (kinetic *K*_*D*_) was calculated to be 0.78 nM.

To obtain kinetic binding parameters for unlabeled A_2B_AR antagonists, a radioligand competition association assay was developed. The specific binding of [^3^H]PSB-603 was measured in the absence and presence of unlabeled PSB-603 over a time course of 45 min (Fig. 1C) and *k*_*on*_, *k*_*off*_ and kinetic *K*_*D*_ values of unlabeled PSB-603 were calculated to be 0.109 nM^−1^ min^−1^, 0.084 min^−1^ and 0.77 nM, respectively (Table 1). As the values of the competition association assay were in excellent agreement with the ones from the association and dissociation assay (Table 1), the first was deemed validated for determining an unlabeled ligand’s binding kinetics.

In order to increase the throughput of the assay, a single concentration of PSB-603 (1.0-fold its IC_50_) was tested. Association and dissociation rate constants were found to be similar to the aforementioned ones, i.e. 0.111 ± 0.014 nM^−1^ min^−1^ and 0.086 ± 0.007 min^−1^ for *k*_*on*_ and *k*_*off*_ respectively (data not shown). Consequently, all other compounds were tested only at one concentration equal to 1.0-fold their IC_50_ determined from displacement experiments.

#### 3.2.2. Determination of equilibrium binding affinity (K_i_ values) of A_2B_AR antagonists

Once the necessary assays were developed and validated, various xanthine-based A_2B_AR antagonists were examined. The affinities of all compounds were evaluated in an equilibrium radioligand displacement study using [^3^H]PSB-603 as the radiolabeled competitor. All compounds fully displaced the radioligand from the hA_2B_ receptor in a concentration-dependent manner. The data were fitted in a one-phase competition model showing mono-phasic displacement. A wide spread of affinities was noticed, ranging from 61.4 μM for compound **4** to 1.78 nM for compound **13**; all affinities are listed in Tables 2–4.

#### 3.2.3. Evaluation of kinetic binding parameters (k_on_, k_off_, RT) of A_2B_AR antagonists

Any compound with a K_i_ value < 100 nM was assessed in kinetic binding assays, i.e. competition association assay. The *k*_*on*_ values of the 15 qualifying compounds exhibited a 26-fold range, spanning from 0.0014 nM^−1^ min^−1^ for **14** to 0.036 nM^−1^ min^−1^ for **3**. On the contrary, *k*_*off*_ values displayed a greater 94-fold variation, with compounds **17** and **3** defining the lower and upper limits, i.e. 0.011 min^−1^ and 1.071 min^−1^, respectively.

#### 3.2.4. Evaluation of ligand binding recovery with a washout assay

**Fig. 2.**
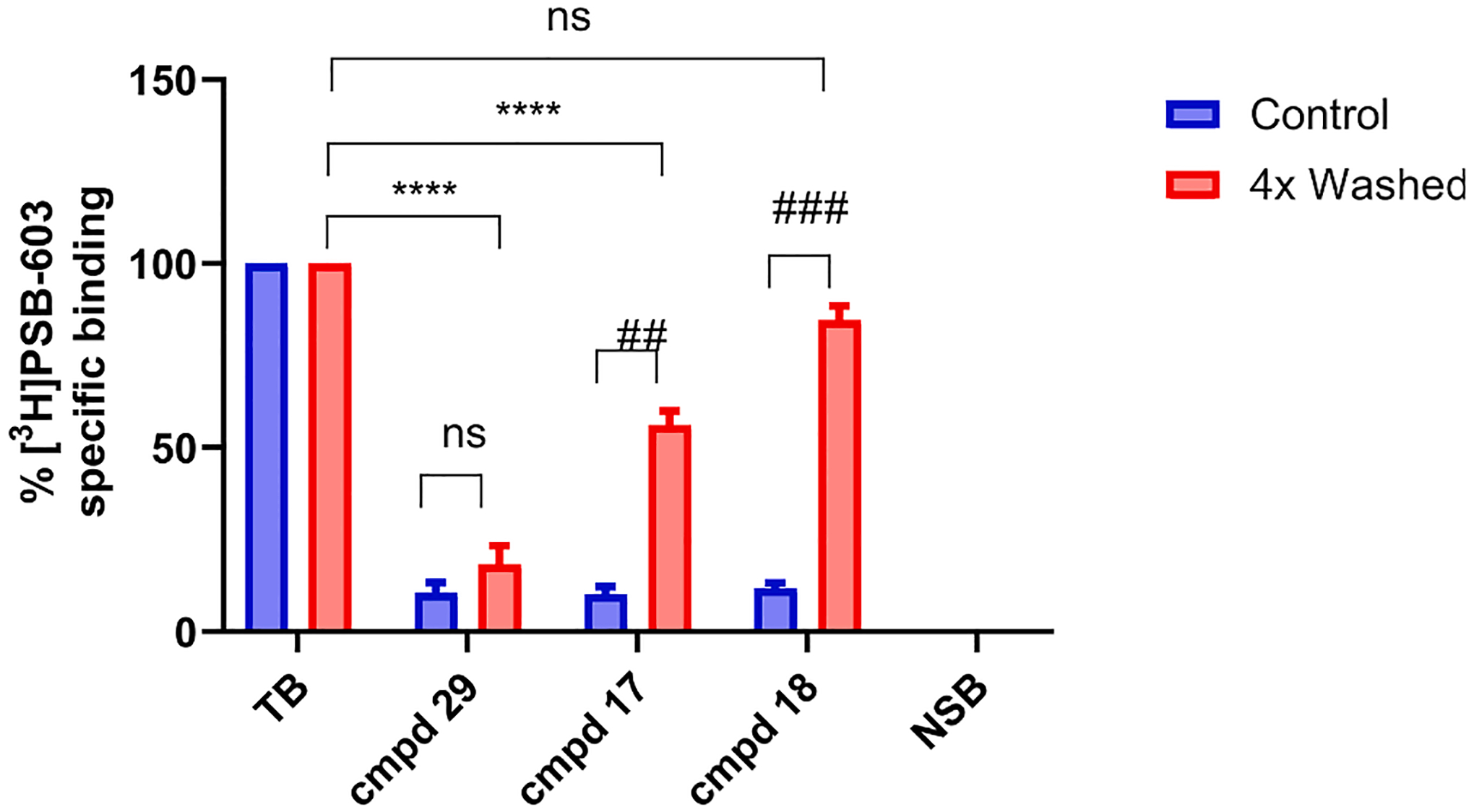
Washout assays of compounds **17**, **18** and **29** at 25 °C. Recovery of radioligand binding in the presence of 10xIC_50_ concentration of **17**, **18** or **29** after no (unwashed) and 4x (washed) washing. Data are shown as mean ± S.E. M. from at least three independent experiments performed in duplicate. The TB and NSB were normalized to 100% (approx. 8000 dpm) and 0% (approx. 2000 dpm) for each condition (control and 4x washed). One-way ANOVA was used for the multiple comparisons of 4x washed compounds to TB. ****p < 0.0001 *t*-test with Welch post-test was used for comparison between control and 4x washed condition for each compound. ##p < 0.01 ###p < 0.001.

To validate the results of the competition association assay and distinguish between ligands with distinct kinetic binding parameters, a [^3^H]PSB-603 washout assay was developed (Fig. 2, Table 5). Compounds **18** and **17** were selected as they presented a short (8 min) and long (87 min) RT compound, respectively, while they have similar structures and affinities. Compound **29**, designed as a putative covalently binding antagonist was also tested in this assay.

Both the washed and the unwashed conditions were assessed. For the washed condition, the unlabeled compounds were incubated with the target for 2 hr, followed by four wash and centrifugation cycles. Subsequently, [^3^H]PSB-603 was co-incubated which led to competition of the radioligand with the unlabeled ligand still bound after the washing procedure. For the unwashed condition no washing was performed before the determination of radioligand displacement. Based on the experimental set-up, the long RT compound and the covalent ligand would be predicted to remain bound to A_2B_ARs, as they would not be easily removed during the washing steps, and thus to result in lower radioligand binding.

In the unwashed condition, all A_2B_ARs were almost fully occupied by each of the compounds as there was little specific binding of [^3^H]PSB-603 observed (Fig. 2; control/unwashed). When the washed and unwashed conditions for both compounds **17** and **18** (Fig. 2; washed and unwashed) were compared, a significant increase in radioligand binding after washing was monitored, indicating that they had (partially) dissociated from the target and been washed away. This was hardly the case for compound **29**. The short RT compound **18** did not show any significant difference in specific [^3^H]PSB-603 binding compared to control (TB). Apparently, **18** was almost completely (85%) removed during the washing procedure. On the other hand, the long RT compound **17** was washed away for 56%, indicating that 44% of A_2B_ARs were still occupied by this ligand after the applied washing cycles, showing a significant (*P <* 0.0001, Fig. 2, washed) decrease of [^3^H]PSB-603 specific binding compared to control (TB). This was even more apparent with compound **29**, with only 18% of material being washed off the receptor.

#### 3.2.5. Structure-Affinity Relationships (SAR) and Structure-Kinetic Relationships (SKR)

We started with the study of the prototypic A_2B_AR antagonist MRS1754 (**13**). In the displacement assay an affinity of 1.78 nM was determined. The kinetic characterization of **13** resulted in a determined RT of 69 min, which made us increase the duration of the competition association assay from 45 min to 3 h for all compounds in order to allow for the longer RT compounds to reach equilibrium.

We initiated the investigation on the xanthine scaffold with compound **1** [24]. Its affinity was found to be higher than 100 nM, the limit set as threshold for the kinetic studies. By substitution of the acid moiety for an acetamide (**2**) the affinity increased approx. 7-fold. Therefore, this acetamide was incorporated in all other compounds synthesized and tested. For compound **2**, it was not possible to determine its kinetic characteristics, most probably because they were outside the detection range of our method.

Further functionalization of the acetamide to incorporate a cyclopropyl group (**3**) decreased the affinity, an effect observed for every non-aromatic ring tested (**3**, **4**, **5**, **7**). When pyrazole was incorporated (**6**) the affinity increased compared to compound **5**, but it remained in the micromolar range. To the contrary, introduction of pyridine (**9**) and pyrazine (**10**) decreased affinity even further when compared to the cyclohexyl substitution (**7**). Only incorporation of a phenyl ring (**8**) resulted in a low nanomolar affinity (1.93 nM) and a RT of 46 min. In addition to the cyclic substituents, a linear one (**11**) was incorporated, leading to a decrease in affinity compared to compound **2**. However, the affinity did not exceed the 100 nM threshold.

Taking these results into consideration we continued with *para* substitution of the phenyl ring and determined the influence of those substituents on affinity and kinetic binding parameters. When we substituted compound **8** with a p-methyl group (**12**) a slight decrease in affinity and a 10 min increase in RT were observed. Introduction of a pcyano group (**13**) increased RT further, while the association rate constant increased about 4 times, yielding a compound with (sub)nanomolar affinity. The introduction of other electron withdrawing groups at the p-position (**14**, **15**, **16**, **17**, **18**) also yielded high affinity values for the receptor. Introduction of a nitro (**14**) or methyl ketone (**16**) substituent resulted in a moderate RT of 58 and 54 min, respectively, while the *k*_*on*_ value was largely varying, with **14** presenting a slow association to the receptor. The trifluoromethyl substituent (**15**) resulted in a high affinity for the receptor, while its kinetic characteristics could not be monitored due to the detection range of the assay. Introduction of a carboxylic acid (**17**) was responsible for the longest RT measured in this study, while a methylcarboxamide (**18**) resulted in a similar affinity but significantly shorter RT.

Subsequently, the introduction of a spacer between the acetamide and the phenyl ring was investigated (Table 4). By introducing a carbon linker (**19**) to compound **8**, the affinity dropped to a value in the micromolar range. A similar trend was observed for compounds **12** and **20**. Although affinity was decreased by 3-fold, it still remained in the nanomolar range for both compounds (**12** and **20**) allowing their kinetic characterization. The *k*_on_ value slightly decreased (0.007 nM^−1^ min^−1^ and 0.004 nM^−1^ min^−1^ for **12** and **20**, respectively), while RT was lessened by about 3-fold for **20**. Substitution of 4-methyl (**20**) by 4-fluoro (**21**), 4-bromo (**22**) and 3,4-di-hydroxy (**23**) did not lead to significant alteration of affinity, while RT remained to be 20 min or less. Only **21** yielded an increased *k*_*on*_ value, hence a faster association to hA_2B_AR. Furthermore, the linker was altered by a methyl substitution on the R^2^ position. The two enantiomers, R (**24**) and S (**25**), exhibited similar affinities and kinetic characteristics, with **24** showing approx. 2-fold increased *k*_*on*_ and *k*_*off*_ values compared to **25**, although the RT was short in both cases. When a phenyl ring was introduced on the R^2^ position (**26**), the affinity slightly dropped compared to **24** and **25**, which after kinetic analysis appeared due to a decrease in association rate constant. The benzyl substitution of the amido group (**27)** resulted in an increase in affinity compared to the unsubstituted **19**, indicating that this benzyl moiety is well accommodated in the binding pocket of A_2B_AR. However, when compared to **26**, **27** showed a lower affinity, suggesting that **27** did not optimally fit. Finally, **29**, bearing a reactive warhead, did not allow the determination of true equilibrium affinity values; its apparent affinity under the conditions tested was approx. 9 nM.

#### 3.2.6. Correlation plots

**Fig. 3.**
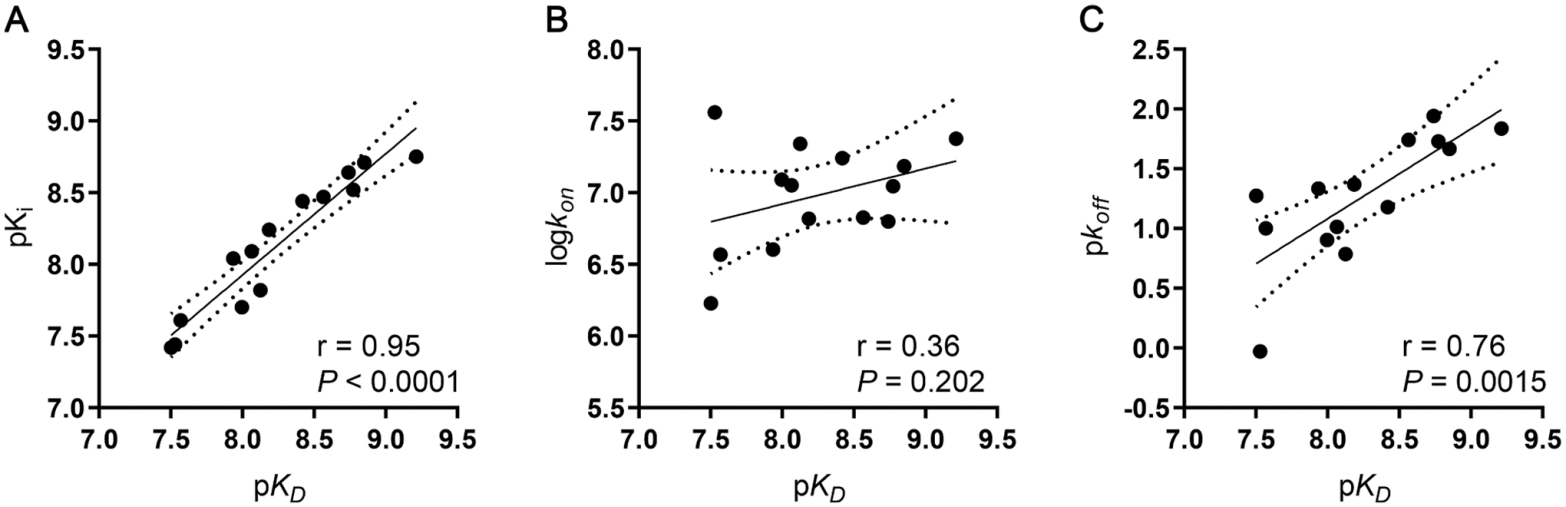
Correlation plots between affinity and kinetic parameters. Kinetic affinity (p*K*_*D*_) is plotted against (A) affinity determined from typical displacement assays (pK_i_); (B) association rate constant (log*k*_*on*_); (C) dissociation rate constant (p*k*_*off*_). The solid line corresponds to the linear regression of the data and the dotted lines represent the 95% confidence intervals for regression. Correlation was tested with the Pearson r coefficient, while significance is shown with a *P* value. Data used in the plots are from Tables 2–4. Data are expressed as mean from at least three independent experiments performed in duplicate.

To obtain a better comparison of kinetic and affinity parameters and understand their relationship, correlation plots for all compounds with measurable rate constants were constructed (Fig. 3). The affinity obtained from traditional radioligand displacement assay (pK_i_) and the kinetic affinity (p*K*_*D*_) derived from the radioligand competition association assay were found to be significantly and strongly correlated (r = 0.95, *P <* 0.0001; Fig. 3A), validating the use of the competition association assay. When the association rate constants (log *k*_*on*_) of all kinetically characterized compounds were plotted against the kinetic affinity (Fig. 3B), a low, non-significant correlation was observed (r = 0.36, *P* = 0.202). However, the kinetic affinity was found to be significantly correlated with the dissociation rate constants (p*k*_*off*_) (r = 0.76, *P* = 0.0015) (Fig. 3C).

#### 3.2.7. Functional characterization of compounds 17 and 18

Next to binding parameters we studied compounds **17** and **18** in a functional set-up, in order to investigate the link between binding kinetics and a possibly prolonged functional effect. For this purpose a label-free assay was developed as described in Materials and Methods and below.

**Fig. 4.**
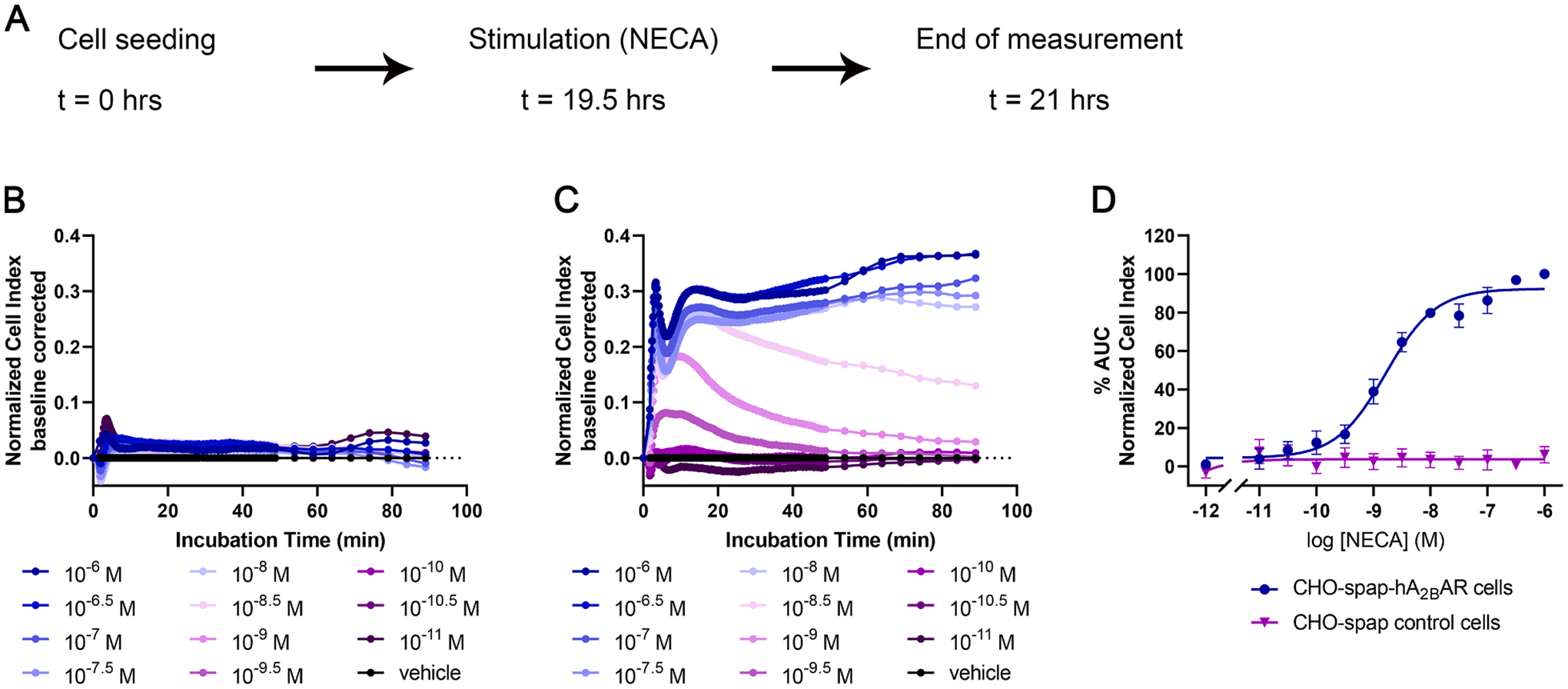
Functional characterization of NECA on CHO-spap and CHO-spap-hA_2B_AR cells. (A) Graphic representation of assay set-up. Cell were seeded and 19.5 h later they were stimulated with NECA (10 pM – 1 μM) and the cell response was monitored for 1.5 h. Representative responses induced by NECA on (B) CHO-spap and (C) CHO-spap-hA_2B_AR cells. (D) Concentration-response curves of NECA. The curves were normalized to minimum (0%) to maximum response (100%) of CHO-spaphA_2B_AR cells. Data shown are mean ± S.E.M. from at least three separate experiments performed in duplicate.

**Fig. 5.**
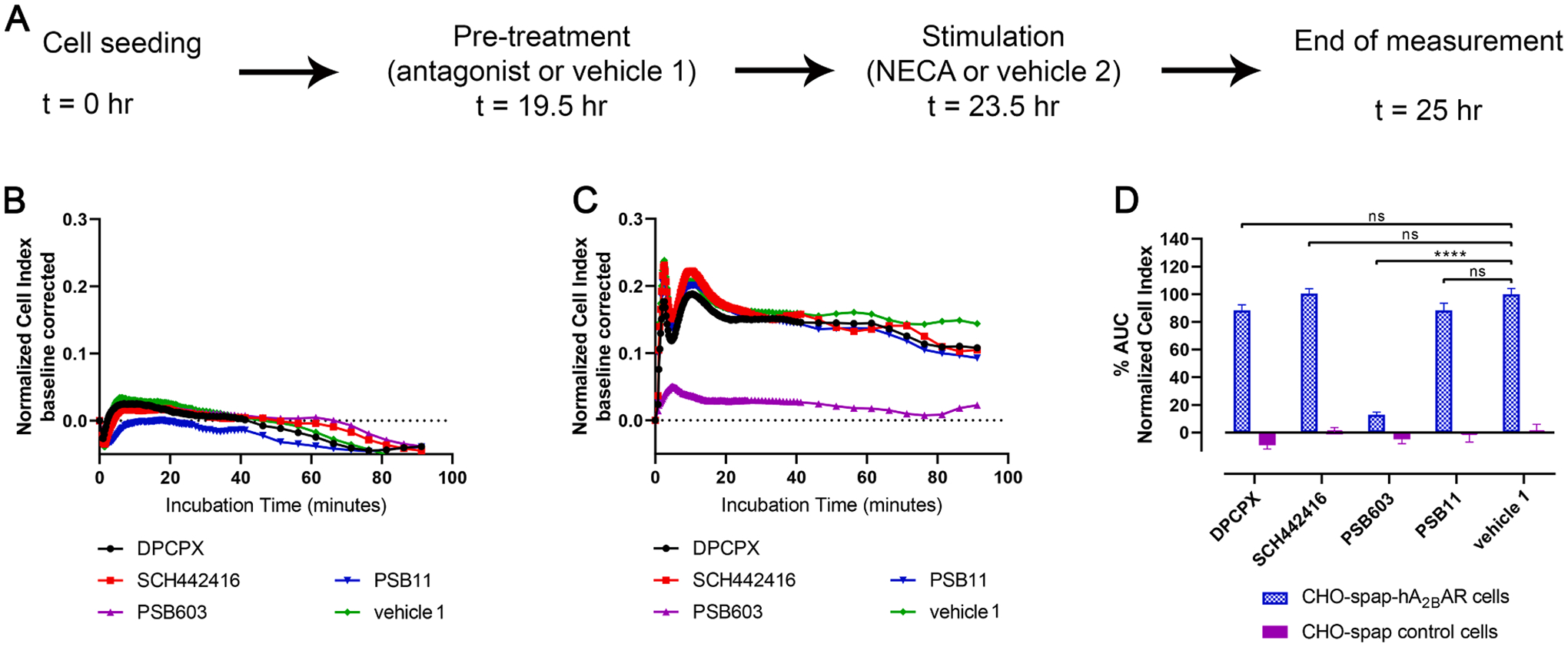
NECA signaling on CHO-spap and CHO-spap-hA_2B_AR cells is only mediated via hA_2B_AR. (A) Graphic representation of assay set-up. Cells were seeded, and 19.5 h later they were pre-treated with an antagonist for all ARs (A_1_: DPCPX; A_2A_: SCH442416; A_2B_: PSB603; A3: PSB11). Later they were stimulated with an EC_80_ concentration of NECA (4 nM) and the cell response was monitored for 1.5 h. Representative responses induced by NECA on (B) CHO-spap and (C) CHO-spap-hA_2B_AR cells. (D) Bar graphs represent the AUC of antagonists for all ARs after stimulation with EC_80_ of NECA. The data were normalized to vehicle 1 treated with NECA of CHO-spap-hA_2B_AR cells as 100%. Data shown are mean ± S.E.M. from at least three separate experiments performed in duplicate. ns *P >* 0.05, *** *P* ≤ 0.001 determined in a one-way ANOVA test with Dunnett’s correction.

Initial experiments with NECA (5^′^-N-ethylcarboxamidoadenosine), a non-selective AR agonist, were performed on control CHO-spap and CHO-spap-hA_2B_ARs cells (Fig. 4). No response was found on “empty” CHO-spap cells upon treatment with NECA, whereas a concentration-dependent response was measured on CHO-spap-hA_2B_ARs cells, yielding a pEC_50_ value of 8.95 (Table 5). In order to validate that the response measured on CHO-spap-hA_2B_ARs was only A_2B_AR mediated, we pre-incubated cells with selective antagonists for each AR subtype prior to NECA stimulation. Only PSB-603, the A_2B_AR-selective antagonist, inhibited the NECA response, confirming the A_2B_AR specific hypothesis (Fig. 5A–D). As a result, further experiments for the study of A_2B_AR were performed on CHO-spap-hA_2B_AR cells.

**Fig. 6.**
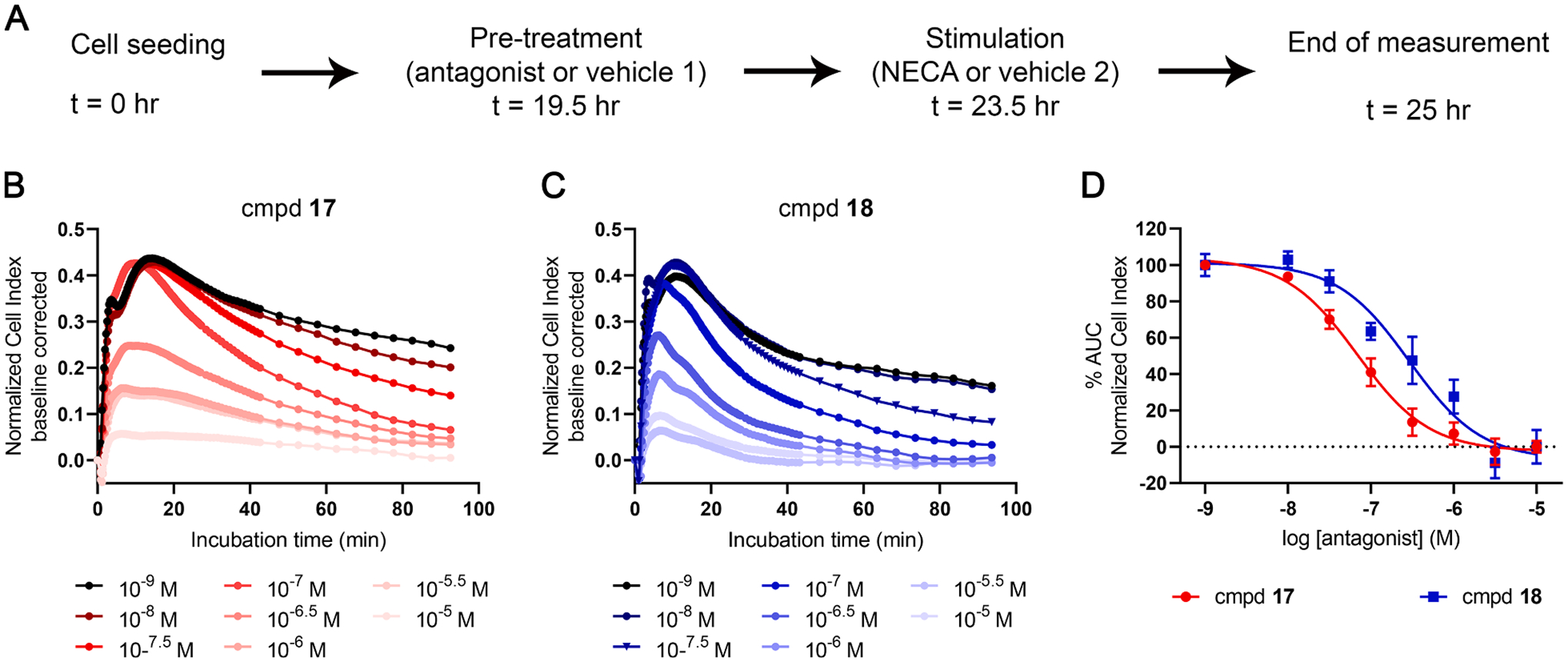
Functional characterization of compound **17** (short RT) and **18** (long RT) on CHO-spap-hA_2B_AR cells. (A) Graphic representation of assay set-up. Cells were seeded, and 19.5 hr later they were pre-treated with antagonist **17** or **18** (1 nM – 10 μМ) or control (vehicle 1; 0.25% DMSO). After a 4 hr incubation, cells were stimulated with an EC_80_ concentration of NECA (4 nM) or control (vehicle 2; 0.25% DMSO) and the cell response was monitored for 1.5 hr. (B, C) Representative responses induced by NECA after pre-treatment with various concentrations of compound **17** (B) and compound **18** (C). (D) Concentration-response curves of antagonists after stimulation with EC_80_ concentration of NECA. The curves were normalized to minimum (0%) and maximum response (100%). Data shown are mean ± S.E.M. from at least three separate experiments performed in duplicate.

After this assay development, chemically similar compounds **17** and **18** but with an 11-fold difference in RT, were selected for further experiments. First, their inhibitory potency in the presence of an EC_80_ concentration of NECA was determined resulting in pIC_50_ values of 7.12 ± 0.13 and 6.44 ± 0.21, respectively (Fig. 6A–D, Table 5). These potencies were found to be approximately 1.5 log unit lower than their affinities determined in the radioligand binding studies (8.64 ± 0.05 and 7.70 ± 0.06 for **17** and **18**, respectively; Table 3), in line with the presence of a high agonist concentration (i.e. EC_80_) in this assay set-up.

**Fig. 7.**
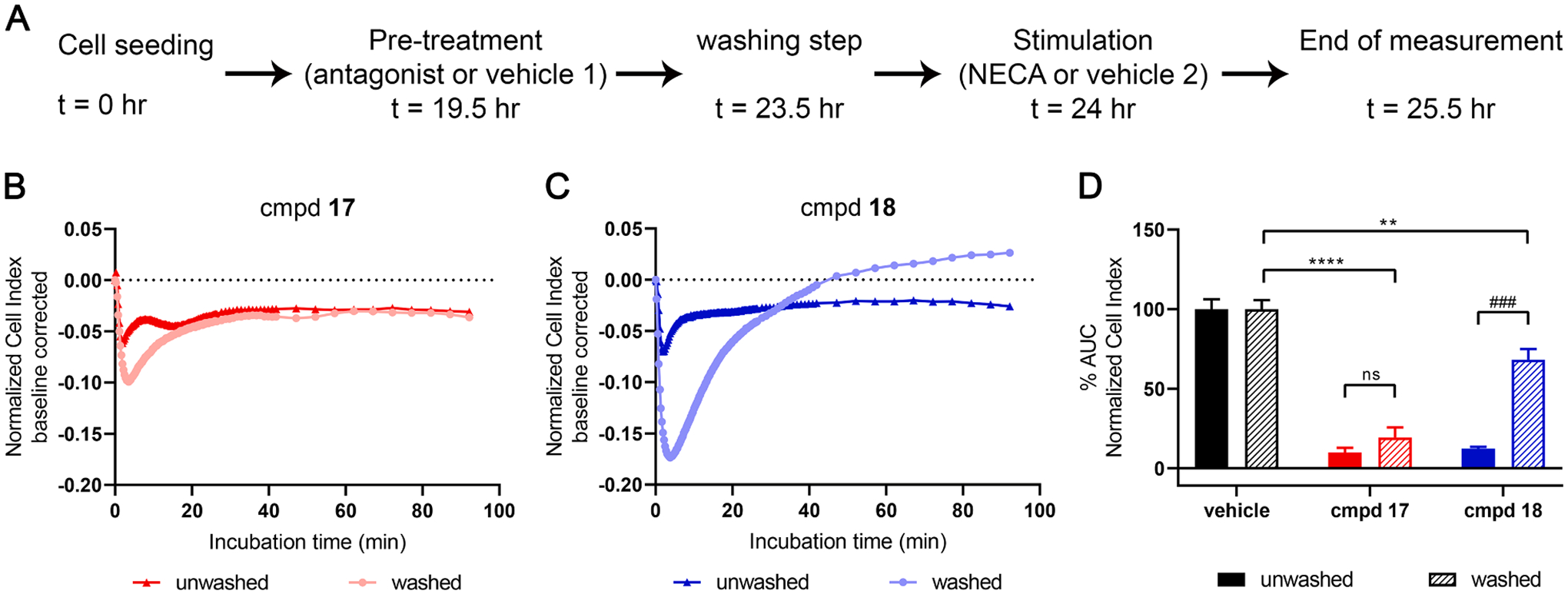
Recovery of NECA signaling after washing. (A) Graphic representation of assay set-up. Cells were seeded, and 19.5 hr later they were pre-treated with an antagonist (30 × IC_50_ as determined in the label-free assay in Fig. 6) or control (vehicle 1; 0.25% DMSO). After a 4 hr incubation, cells were washed by removing the medium from the well and replacing it with 95 μL of fresh serum-free medium. For the unwashed condition, no medium refreshment was done. 30 min afterwards, cells were treated with NECA (at an EC_80_ concentration) or control (vehicle 2; 0.25% DMSO) and the cell response was monitored for 1.5 hr. (B,C) Representative responses induced by NECA with or without washing of cells pre-treated with compound **17** (B) and compound **18** (C). (D) Bar graph showing NECA response after washing step when cells were pre-treated with compound **17** or **18**. Bar graphs of both washed and unwashed conditions were compared to the control (vehicle) without any antagonist (100% washed and unwashed AUC, respectively). Data shown are mean ± S.E.M. from at least three separate experiments performed in duplicate. ns *P >* 0.05, ^# # #^ *P* ≤ 0.001 determined in an unpaired *t*-test with Welch’s correction. ** *P* ≤ 0.01, **** *P* ≤ 0.0001 determined in a one-way ANOVA test with Dunnett’s correction.

Subsequently, a washout assay was performed and the cell response to the compounds was monitored and evaluated (Fig. 7). In short, cells were pre-treated with a concentration of 30 × IC_50_ of the compounds, and 4 hr later cells were washed, and fresh”serum-free medium” was added (Fig. 7A). For the evaluation of the unwashed condition, the medium was not refreshed but was pipetted up and down in order to mimic the possible mechanical stress induced to the washed cells. Cells were then stimulated with an EC_80_ concentration of NECA, enabling us to monitor the response exerted by only those receptors that were not bound to compounds **17** (Fig. 7B) and **18** (Fig. 7C). Based on the experimental set-up, it was hypothesized that the short RT compound was removed more readily during washing, resulting in an increased number of receptors available for NECA to bind and cause a cellular response.

Cells pre-treated with long RT compound **17** showed no significant increase (p > 0.05) in NECA signaling after washing (9.8% and 12% for unwashed and washed cells, respectively) (Fig. 7D, Table 5). On the contrary, the increase in NECA signaling between unwashed and washed cells was significantly higher (*P <* 0.001) with the short RT compound **18** (19% and 68% for unwashed and washed cells, respectively) (Fig. 7D, Table 5), verifying our hypothesis. This assay simulates a non-equilibrium condition, not unlike human physiology.

#### 3.2.8. Binding affinity of selected compounds at other adenosine receptors

Selected xanthine derivatives were compared in binding assays [24] at four adenosine receptors (human and rat) as shown in Table 6. The A_2B_ receptor selectivity was generally low, especially at the rat A_2B_ receptor. However, several derivatives, e.g., **20** and **22**, displayed mixed higher affinity at the human A_2B_ and A_2B_ receptors compared to the other subtypes.

## 4. Discussion

Intrigued by earlier studies [34] we combined previously synthesized xanthines with a series of newly synthesized derivatives. In this way we obtained a total of 28 final compounds that were subsequently tested in a variety of assays. The aim was to analyze both their structure-affinity and structure-kinetics relationships, and to test the translational properties of two selected derivatives in a number of label-free assays. We had performed similar studies on other GPCRs before, and learned that the sole determination of (equilibrium) affinity values provides an incomplete profile of the pharmacological characteristics of receptor ligands. Adding kinetic information informed us better, and made clear that target binding kinetics can dictate the duration of action and the sustainability of a pharmacological effect, thus having an impact on the ligand’s pharmacokinetic and pharmacodynamic behavior *in vivo* [35].

We first tested the behavior of [^3^H]PSB-603, the radioligand used in the present study, and learned that the K_D_ value we derived was comparable to the value previously reported by Bormann *et al*. [36] A similar K_D_ value was derived from the kinetic association and dissociation experiments, providing a reliable framework for our further experiments. We then used the radioligand to determine the affinity of the compounds for the hA_2B_AR. Some of the previously synthesized xanthines had been tested on a number of other human and rat ARs (Table 6), showing a limited selectivity at best. It is important to stress that selectivity was not pursued in the current study.

We then determined the target binding kinetics of the full series of xanthine derivatives following an established protocol by Motulsky and Mahan [32]. This so-called competition association assay has been used to investigate the binding kinetics of ligands for various targets, such as GPCRs [37], kinases [38], transporters [39,40] and other proteins [41]. As the values from the competition association assay were in excellent agreement with the ones from the association and dissociation assay (Table 1), the first was deemed validated for determining an unlabeled ligand’s binding kinetics. Evaluation and incorporation of target binding kinetics has been found to be a crucial parameter in drug optimization. The association rate constant is crucial for high target occupancy, due to the resulting rebinding effect [42], as well as for drug selectivity over different targets and ultimately for increased drug safety [43]. Last but not least, a fast association is crucial for an immediate drug response in case of an acute pathological event [44,45]. As far as the dissociation rate constant is concerned, a slow rate, hence a long RT is required for a longer and/or a more durable, sustained effect [46]. If RT exceeds the pharmacokinetic half-life, the drug could maintain its effect even past plasma clearance, resulting in potential advantages like a decreased frequency of drug dosing and a reduction in off-target toxic effects [43,47]. The compounds showed largely varying equilibrium affinity values as well as kinetic rate constants and corresponding residence times (RTs). Among the compounds was the prototypic A_2B_AR antagonist MRS1754 (**13**). In the displacement assay an affinity of 1.78 nM was determined for **13**, which was in excellent agreement with data reported by Ji *et. al*. [48]. The kinetic characterization of **13**, yielding an RT of 69 min, made us increase the duration of the competition association assay from an initial 45 min to 3 h in order to allow for the longer RT compounds to reach equilibrium. As a result the 3 hr incubation assay was used for the determination of kinetic binding parameters for all qualifying A_2B_AR antagonists. Compound **3** had the shortest RT (0.9 min), while compound **17** was endowed with the longest (87 min). Overall, there was a significant correlation between affinity and dissociation rate constants of all compounds for which we were able to obtain the kinetic parameters (Fig. 3C).

Due to its high chemical similarity we chose compound **18** (RT = 8.0 min) as a benchmark comparator for compound **17**. We performed radioligand washout experiments with both compounds and compared their behavior with that of a putatively covalent antagonist (**29**). It appeared that **18** was rapidly dissociating from the receptor under washing conditions, while **17** was much more resistant, although less so than **29**. Currently, A_2B_AR antagonists are in clinical stages of testing for their immuno-oncological behavior (*vide supra* and ref [14]). The tumor micro-environment where the compounds supposedly act is under the influence of high adenosine levels. Hence antagonists with longer RT, counterbalancing this adenosine “pressure”, may be very useful.

Next to binding parameters we studied compounds **17** and **18** in a functional set-up, in order to investigate the link between binding kinetics and a possibly prolonged functional effect. For this purpose a so-called label-free assay was developed with xCELLigence technology, measuring changes in cellular impedance that are expressed as a unitless parameter named Cell Index [28,30]. As mentioned before we found a relatively high potency [49,50] for the reference, non-selective, AR agonist NECA, although it complies with other data in literature [51–54]. Hence, we made sure that the effects seen were entirely due to interactions with the A_2B_AR. The same assay was used to study wash-out of the two compounds, simulating a non-equilibrium condition not unlike human physiology. As a result, findings from this assay, with **17** largely maintaining its effect, constitute a possible translational step towards *in vivo* experiments [27,55].

In conclusion we reported the synthesis and pharmacological evaluation of a series of xanthine-based analogues designed as hA_2B_AR antagonists. A radioligand competition association assay was developed to evaluate kinetic binding parameters next to affinity. Structure-affinity and structure-kinetic relationships (SAR and SKR) were examined and a great spread in target residence time (RT) was observed, from 0.9 min (**3**) to 87 min (**17**). Based on correlation plots, the dissociation rate constant appeared the driving force for affinity unlike the association rate constants. Subsequently two compounds (**17** and **18**) with long and short RT, respectively, were selected and tested in a label-free impedance-based assay. These experiments confirmed the link between long RT and an extended pharmacological effect under non-equilibrium conditions. To our knowledge, this is the first SKR study performed on hA_2B_AR antagonists, which could pave the way to the development of clinically meaningful antagonists, e.g., in immune-oncology, with a high affinity and long residence time at the A_2B_AR.

## Figures and Tables

**Table 1:** Comparison of the affinity, dissociation constants and kinetic parameters of PSB-603, obtained with different radioligand binding assays performed on CHO-spap-hA_2B_AR membranes.

Assay	B_max_ (pmol/mg)	*K*_*D*_ (nM)	pK_i_ (K_i_ (nM))	*k*_*on*_ (nM^−1^ min^−1^)	*k*_*off*_ (min^−1^)	RT (min)
Saturation binding	4.30 ± 0.30	1.71 ± 0.14	–	–	–	–
Displacement	–	–	8.90 ± 0.10 (1.25)	–	–	–
Association and dissociation	–	0.78 ± 0.09^a^	–	0.096 ± 0.010	0.075 ± 0.003	13 ± 0.6
Competition association	–	0.77 ± 0.10^a^	–	0.109 ± 0.008^b^	0.084 ± 0.009^b^	12 ± 1.3

Values are mean ± S.E.M. of at least three individual experiments performed in duplicate.

a Kinetic affinity values (K_D_) determined by association and dissociation, and competition association assay, is defined as *K*_*D*_ = *k*_*off*_ */k*_*on*_.

b Kinetic parameters of unlabeled PSB-603 were determined by addition of 0.3-, 1- and 3-fold its IC_50_ value.

**Table 2:** Affinity (pKi) and Kinetic Parameters (*k*_*on*_, *k*_*off*_, RT) of hA_2B_AR antagonists **1** – **11**.

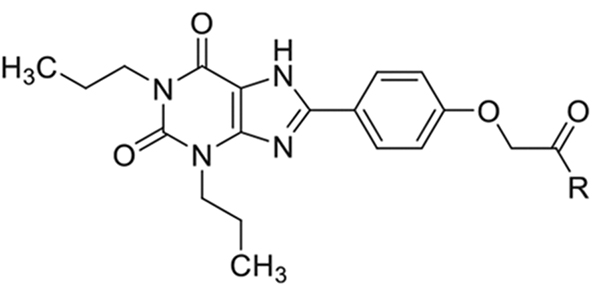					
cmpd	R	pKi (Ki (nM))	*k*_*on*_ (nM^−1^ min^−1^)	*k*_*off*_ (min^−1^)	RT^a^ (min)
**1**	OH	6.78 ± 0.06 (167)	n.d.^b^	n.d.	n.d.
**2**	NH_2_	7.60 ± 0.07 (25.1)	Kinetics outside the range of the assay (see text)		
**3**	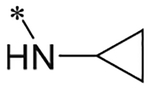	7.44 ± 0.09 (36.3)	0.036 ± 0.006	1.071 ± 0.027	0.9 ± 0.0
**4**	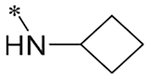	4.21 ± 0.14 (61423)	n.d.	n.d.	n.d.
**5**	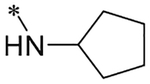	4.78 ± 0.17 (16749)	n.d.	n.d.	n.d.
**6**	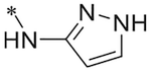	5.26 ± 0.05 (5483)	n.d.	n.d.	n.d.
**7**	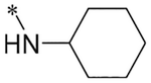	6.02 ± 0.09 (951)	n.d.	n.d.	n.d.
**8**	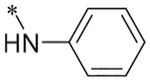	8.71 ± 0.01 (1.93)	0.015 ± 0.000	0.022 ± 0.006	46 ± 13
**9**	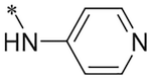	5.87 ± 0.16 (1351)	n.d.	n.d.	n.d.
**10**	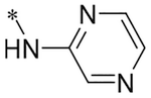	5.16 ± 0.08 (6950)	n.d.	n.d.	n.d.
**11**	N (CH_3_COOEt)_2_	6.95 ± 0.00 (1 1 3)	n.d.	n.d.	n.d.

Values represent the mean ± S.E.M. of at least three individual experiments, performed in duplicate.

a RT = 1/*k*_*off*_.

b n.d. = not defined.

**Table 3:** Affinity (pKi) and Kinetic Parameters (*k*_*on*_, *k*_*off*_, RT) of hA_2B_AR antagonists **12** – **18**, **29**.

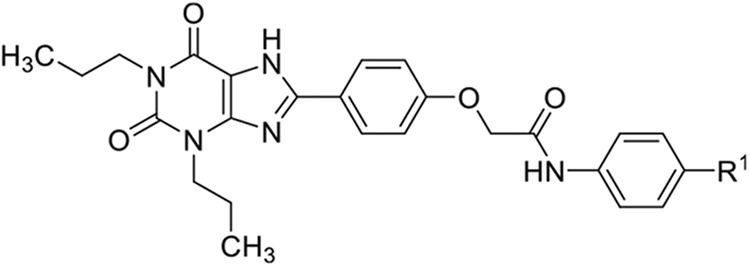						
cmpd	R^1^	pKi (Ki (nM))	*k*_*on*_ (nM^−1^ min^−1^)	*k*_*off*_ (min^−1^)	RT^a^ (min)	*K*_*D*_^b^ (nM)
**12**	CH_3_	8.47 ± 0.06 (3.42)	0.007 ± 0.002	0.018 ± 0.008	55 ± 25	2.7 ± 1.5
**13**	CN	8.75 ± 0.20 (1.78)	0.024 ± 0.005	0.015 ± 0.001	69 ± 2.4	0.61 ± 0.14
**14**	NO_2_	7.87 ± 0.04 (13.5)	0.0014 ± 0.0003	0.017 ± 0.006	58 ± 19	12 ± 4.9
**15**	CF_3_	8.54 ± 0.06 (2.86)	Kinetics outside the range of the assay (see text)			
**16**	COCH_3_	8.52 ± 0.05 (3.03)	0.011 ± 0.001	0.019 ± 0.006	54 ± 18	1.7 ± 0.58
**17**	COOCH_3_	8.64 ± 0.05 (2.29)	0.006 ± 0.001	0.011 ± 0.004	87 ± 29	1.8 ± 0.62
**18**	CONHCH_3_	7.70 ± 0.06 (19.9)	0.012 ± 0.001	0.125 ± 0.003	8.0 ± 0.2	10 ± 0.89
**29**	NCS	8.03 ± 0.05 (9.28)	Covalent mechanism (see text)			

Values represent the mean ± S.E.M. of at least three individual experiments, performed in duplicate.

a RT = 1/*k*_*off*_.

b Kinetic *K*_D_ values, defined as *K*_D_ = *k*_*off/*_*/k*_*on*_.

**Table 4:** Affinity (pK_i_) and Kinetic Parameters (*k*_*on*_, *k*_*off*_, RT) of hA_2B_AR antagonists **19** – **27**.

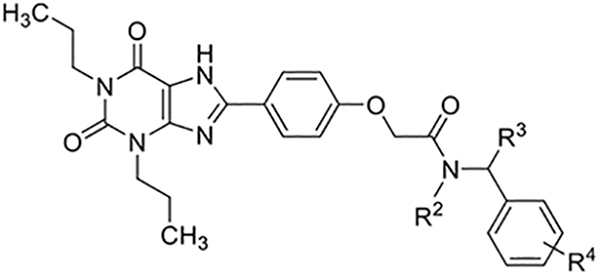								
Cmpd	R^2^	R^3^	R^4^	pKi (Ki (nM))	*k*_*on*_ (nM^−1^ min^−1^)	*k*_*off*_ (min^−1^)	RT^a^ (min)	*K*_*D*_^b^ (nM)
**19**	H	H	H	6.15 ± 0.15 (711)	n.d.^c^	n.d.	n.d.	n.d.
**20**	H	H	4-CH_3_	8.04 ± 0.04 (9.08)	0.004 ± 0.0015	0.046 ± 0.007	22 ± 3.5	12 ± 4.7
**21**	H	H	4-F	8.44 ± 0.06 (3.60)	0.017 ± 0.007	0.066 ± 0.015	15 ± 3.5	3.8 ± 1.7
**22**	H	H	4-Br	8.24 ± 0.11 (5.73)	0.007 ± 0.002	0.043 ± 0.019	23 ± 11	6.5 ± 3.6
**23**	H	H	3,4-diOH	7.61 ± 0.04 (24.4)	0.004 ± 0.001	0.099 ± 0.011	10 ± 1.1	27 ± 7.9
**24** ^d^	H	CH_3_	H	7.82 ± 0.07 (15.2)	0.022 ± 0.008	0.164 ± 0.042	6.1 ± 1.6	7.5 ± 3.9
**25** ^d^	H	CH_3_	H	8.09 ± 0.04 (8.11)	0.011 ± 0.003	0.097 ± 0.021	10 ± 2.3	8.6 ± 3.0
**26**	H	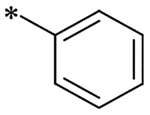	H	7.42 ± 0.18 (37.9)	0.0017 ± 0.0006	0.053 ± 0.017	19 ± 6.2	31 ± 16
**27**	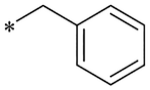	H	H	6.92 ± 0.05 (1 1 9)	n.d.	n.d.	n.d.	n.d.

Values represent the mean ± S.E.M. of at least three individual experiments, performed in duplicate.

a RT = 1/*k*_*off*_.

b Kinetic *K*_D_ values, defined as *K*_D_ = *k*_*off*_ */k*_*on*_.

c n.d. = not defined.

d *R*- (**24**) or *S*- (**25**) enantiomer.

**Table 5:** Potency values (pEC_50_ and pIC_50_) of compounds in a label-free assay, and NECA signaling therein before and after washing, all on CHO-spap-hA_2B_AR cells.

Compound	Label-free assay			
	pEC_50_ (EC_50_ (nM))	pIC_50_ (IC_50_ (nM))	washout - %AUC	
			unwashed	1x washed
**NECA**	8.95 ± 0.13 (1.12)	n.a.	100	100
**17**	n.a	7.12 ± 0.13 (75.4)	10 ± 3	19 ± 6
**18**	n.a.	6.44 ± 0.21 (363)	12 ± 1	68 ± 7

Values represent the mean ± S.E.M. of at least three individual experiments, performed in duplicate. n.a. = not applicable.

**Table 6:** Comparison of adenosine receptor binding affinities of selected antagonists, expressed as mean ± S.E.M (n = 3 – 4). The species is human, unless noted (r, rat).^a^.

Compound	K_i_ (A_1_, nM)	K_i_ (A_2A_, nM)	K_i_ (A_2B_, nM)	K_i_ (A_3_, nM)
**3**	10.7 ± 1.75 (r)	51.7 ± 19.6 (r)	36.6 ± 5.1 (r)	ND
**5**	13.9 ± 1.8 (r)	19.1 ± 5.1 (r)	26.3 ± 192 (r)	ND
**7**	23.0 ± 4.1 (r)	19.3 ± 6.7 (r)	73.5 ± 25.8 (r)	ND
**19**	54.7 ± 21.2, 5.02 ± 0.55 (r)	23.8 ± 5.7, 25.9 ± 7.6 (r)	2.04 ± 0.17, 19.8 ± 7.9 (r)	ND ND
**20**	212 ± 68, 8.85 ± 1.75 (r)	20.8 ± 2.4, 207 ± 26 (r)	8.0 ± 2.2, 486 ± 192 (r)	140 ± 7 ND
**21**	63.9 ± 16.5, 6.55 ± 1.27 (r)	33.6 ± 0.1, 251 ± 33 (r)	5.5 ± 1.2, 20.0 ± 4.9 (r)	89.9 ± 11.6 ND
**22**	586 ± 250, 32.7 ± 1.27 (r)	26.1 ± 0.4, 112 ± 12 (r)	6.8 ± 0.8 ND	274 ± 56 ND
**24**	27.3 ± 4.9, 5.23 ± 1.13 (r)	64.4 ± 21.2, 375 ± 139 (r)	8.5 ± 1.1, 21.1 ± 5.3 (r)	63.9 ± 16.5 ND
**25**	23.7 ± 4.9, 10.4 ± 2.1 (r)	21.0 ± 1.8, 165 ± 53 (r)	8.2 ± 2.5, 20.2 ± 4.9 (r)	156 ± 15 ND

a All receptors are expressed in HEK-293 cells. Radioligands used: A_1_, [^3^H]*R*-PIA; A_2A_, [^3^H]CGS21680; A_2B_, [^125^I]I-ABOPX; A_3_, [^125^I]I-AB-MECA. ND, not determined.

## Abbreviations:

AR: adenosine receptor
BCA: bicinchoninic acid
BSA: bovine serum albumin
CGS21680: 4-[2-[[6-amino-9-(N-ethyl-β-D-ribofuranuronamidosyl)-9H-purin-2-yl]amino]ethyl]benzenepropanoic acid
CHAPS: 3-[(3-cholamidopropyl)dimethylammonio]-1-propanesulfonate hydrate
cmpd: compound
DMSO: dimethyl sulfoxide
GPCR: G protein-coupled receptor
h: human
I-AB-MECA: 3-iodo-4-aminobenzyl-5′-N-methylcarboxamidoadenosine
I-ABOPX: 2-[4-[3-[(4-amino-3-iodophenyl)methyl]-2,6-dioxo-1-propyl-7H-purin-8-yl]phenoxy]acetic acid
NECA: 5^′^-N-ethylcarboxamidoadenosine
NSB: non-specific binding
*R*-PIA: N^6^-*R*-phenylisopropyladenosine
RT: residence time
SAR: structure-affinity relationship
SKR: structure-kinetics relationship
TB: total binding
